# Human iPSC-Derived Brain Cells, Organoids, and Organoids-on-Chips: Advancing Neurological Disease Modeling to Improve Clinical Outcome

**DOI:** 10.3390/cells15191768

**Published:** 2026-09-29

**Authors:** Gabriel Burdman, Marco Hanna, Adriana Yndart Arias, Arti Vashist, Madepalli K. Lakshmana

**Affiliations:** 1 Department of Cellular and Molecular Medicine, Herbert Wertheim College of Medicine, Florida International University, Miami, FL 33199, USA; gburd004@fiu.edu (G.B.); mhann057@med.fiu.edu (M.H.); ayndarta@fiu.edu (A.Y.A.); avashist@fiu.edu (A.V.); 2 College of Arts, Sciences and Education, Florida International University, Miami, FL 33199, USA

**Keywords:** Alzheimer’s disease, amyotrophic lateral sclerosis, Huntington’s disease, Parkinson’s disease, human induced pluripotent stem cells, CRISPR, organoids, assembloids, translational validation

## Abstract

**Highlights:**

**What are the main findings?**

• To improve translational accuracy, modern strategies are shifting from animal testing to human cell models, like iPSC neurons, organoids, spheroids, and organs-on-chips.
• The ability of iPSCs to integrate into host brain networks, differentiate into diverse neural and glial lineages, and drive functional recovery has been validated by several animal studies.

**What are the implications of the main findings?**

• iPSC-derived brain cell types (neurons, astrocytes, microglia-like cells, and oligodendrocytes) hold immense promise for modeling neurological diseases. These models will deepen our understanding of disease mechanisms, drive new drug discovery, and advance cell-based therapies.
• While iPSC technology holds great promise, a primary concern is that reprogramming somatic cells to an embryonic state erases the adult aging phenotype, meaning epigenetic profiles and telomere lengths will not match in vivo adult counterparts.

**Abstract:**

Neurological disorders are the leading cause of overall disease and disability worldwide, affecting more than 3.4 billion people globally and over 180 million Americans. Yet there are no curative treatments available for any of the cardinal neurological diseases, such as Alzheimer’s disease (AD), Parkinson’s disease (PD), amyotrophic lateral sclerosis-frontotemporal dementia (ALS-FTD) and Huntington’s disease (HD). Many compounds that demonstrate excellent activity in preclinical models fail in clinical trials. The most compelling reason for this failure is attributed to inherent limitations of rodent models that fail to replicate complex human biology. The transformation of patient-derived somatic cells into induced pluripotent stem cells (iPSCs) that can become functional human neurons is an emerging technology that is expected to revolutionize medical treatments and personal medicine. Here, we review the available evidence for the efficacy of iPSC-derived neurons, organoids, assembloids and organ-on-chip in models of AD, PD, ALS-FTD and HD. We focus on how these human-relevant iPSC-derived brain cells are crucial for understanding neurological disease pathogenesis and accelerating drug discovery. We also discuss the current limitations of this technology and how alternative models such as induced neurons (iNs) are generated by directly reprogramming fibroblasts that preserve age-associated epigenetic signatures and may serve as better models for neurological disorders.

## 1. Introduction

Despite intensive research and costly clinical trials, the discovery of disease-modifying treatments (DMTs) for cardinal neurological disorders, like Alzheimer’s disease (AD), Parkinson’s disease (PD) and amyotrophic lateral sclerosis (ALS), remains vastly insufficient [1,2,3]. Although an inadequate understanding of the complex pathophysiology of neurological disorders and under-validated target selections are the most probable explanations for the failures, the heavy reliance on non-human preclinical models, such as mice and rats, which fail to replicate complex human neurobiology, protein aggregation, and cell death, is also emerging as a probable cause of clinical failures [4,5,6]. The translational failure is not surprising given that human brains differ fundamentally from rodent brains in size, cortical organization, connectivity with long axons, epigenomic information and high-level functional specialization. Also, many hallmark pathological features of human diseases, such as tau phosphorylation or Aβ oligomer diversity, cannot be fully reproduced in rodents. Consequently, a therapeutic target validated well in rodent models may not translate within the redundant, highly specialized neural networks of the human brain. However, we predict that new and emerging technologies are expected to quickly and significantly advance disease modeling, the understanding of pathogenesis, and drug discovery for neurological disorders. Year 2026 marks two decades since Takahashi Yamanaka [7] and, subsequently, Yu et al. [8] successfully achieved induction of pluripotent stem cells from mouse embryonic and adult fibroblasts by introducing four transcription factors: Oct3/4, Sox2, c-Myc and Klf4 or Nanog and Lin28 in the place of c-Myc and Klf4. Thereafter, other cell types including keratinocytes, hepatocytes and T lymphocytes were also successfully reprogrammed into human induced pluripotent stem cells (iPSCs) [9]. Following these discoveries, there is significant interest and impactful utilization of iPSC-derived neurons for multiple neurological disorders not only as mutation-specific disease models [10,11,12] but also to understand disease pathogenesis [13,14,15,16], drug discovery [17,18,19,20] and, importantly, cell therapy [20,21,22,23,24]. Further advances have also been achieved by utilizing genome-editing technology, like the clustered regularly interspaced short palindromic repeat (CRISPR)/Cas9 system, which enables the introduction of desired gene mutations crucial for the generation of isogenic control cells with the corrected mutation, thereby serving as bona fide controls for comparison of pathological and therapeutic responses [25,26,27,28]. Soon, iPSC-derived brain cell types are likely to impact neurodegenerative diseases, as they have the advantages of easy accessibility, scalability, potential for developing personalized medicine based on specific mutations that contribute to the disease and, most importantly, because they could serve as better human disease models. Thus, a new strategy has been shifting from animal models to human cell models, like iPSC neurons [13,14,15,16], 3D spheroids [29,30,31], organoids [32,33,34] and organs-on-chips [35,36,37], because human cells predict human responses better than animals. In this article, we review the recent advances in modeling human neurological diseases, such as AD, PD, ALS and Huntington’s disease (HD), using iPSCs, spheroids and organoids and further update the role of the CRISPR/Cas9 system and the state of drug discovery efforts using these models. It is also important to note that, in addition to iPSC technology, skin fibroblasts can be directly reprogrammed into induced neurons (iNs) without progressing through iPSCs by the introduction of transcription factors [38,39] or microRNAs [40,41]. We are quite optimistic that iPSC-derived brain cell types such as neurons, astrocytes, microglia-like cells and oligodendrocytes would ultimately be immensely useful in modeling neurological diseases, which in turn is likely to lead to better understanding of disease pathogenesis, new drug discovery and cell-based therapies, as schematically illustrated in Figure 1.

## 2. Alzheimer’s Disease

Alzheimer’s disease (AD) is a devastating neurodegenerative disorder of the elderly, presenting with progressive intellectual deterioration, ultimately leading to total dependence on nursing care [42,43,44]. Globally, more than 50 million people are affected, and the number of patients with AD and other dementias is estimated to increase to 152 million by 2050 [45,46]. There are two forms of AD: familial (fAD), which accounts for less than 5% of cases, and sporadic (sAD), accounting for more than 95% of cases of AD. The vast majority of fAD is associated with mutations within the genes amyloid precursor protein (APP) and presenilin 1 and 2 (PSEN1 and PSEN2) [47,48]. sAD is presumably triggered by the interplay of genetic and environmental factors, with unclear etiology [49]. One of the major risk factors associated with sAD is the APOE4 allele, while the APOE2 allele is considered a protective factor. Apart from APOE, genome-wide association studies have identified more than 90 AD risk genes, and detailed studies of these in the field of iPSCs are beginning to emerge [50,51,52]. The major hallmark features of AD are the deposition of amyloid beta (Aβ) peptides, which tend to aggregate and lead to the formation of Aβ plaques, and the formation of hyperphosphorylated tau (p-Tau) by kinases such as glycogen synthase kinase 3β (GSK-3β) and cyclin-dependent kinase 5 (CDK5); their aggregation in neurons leads to the formation of neurofibrillary tangles. However, the specific mechanisms underlying these hallmark features, and the relationship and inter-dependency between p-Tau and Aβ peptides, remain unclear. Thus, iPSCs carrying these genetic mutations are invaluable cellular models for investigating the etiology of AD [11,13,16,17,51].

### 2.1. Familial Disease: Robust Phenotypes, Bounded Generalizability

Both iPSC-derived brain cells and organoids have been investigated to define the role of fAD genes. As expected, iPSC-derived neurons from a fAD patient with an APP gene mutation (APP-E693Δ) showed Aβ oligomer formation with increased markers of endoplasmic and oxidative stress that could be ameliorated with docosahexaenoic acid (DHA) [53]. In another study, an engineered three-dimensional neural culture showed that extracellular Aβ deposition precedes tau accumulation and that γ-secretase inhibition blocks both [54]. Also, a 2026 matrix model reproduced amyloid, tau, mitochondrial and neuronal phenotypes with γ-secretase rescue and microglial uptake [55]. In addition to iPSC-derived neurons, iPSC-derived microglia [56,57,58] and astrocytes with neuropathological features of AD have been successfully generated [59,60,61]. After substantial efforts to generate oligodendrocytes from iPSCs, which requires several months of time [62,63,64], a recent study [65] established an efficient and reproducible method for generating oligodendrocytes from human iPSCs that faithfully recapitulates key developmental transitions, progressing from NPCs to OLIG2^+^/NKX2.2^+^ oligodendrocytes in a relatively short time.

However, these models are not organoids; both rely on transgene expression, and neither report circuit function nor establish networks capable of transmitting signals. On the contrary, 3D neural organoids are preferred because they are better than 2D cultures and animal models as they more closely recapitulate the in vivo tissues and provide greater physiological relevance and reproducibility with human-specific genetic and physiological features. fAD and Down syndrome organoids reproduce amyloid aggregates, tau phosphorylation and cell death without transgene overexpression [66,67]. There is evidence of AD-related amyloid and apolipoprotein E co-pathologies in trisomy 21 organoids [68], and more sensitive detection of soluble aggregates without a functional or causal test also has been established and studied [69]. Another study showed that organoids generated from *PITRM1*-knockout iPSCs exhibited pathological features of AD, including neuronal cell death, tau pathology, and accumulation of Aβ protein aggregates [70]. Interestingly, another study reported that some PSEN1 mutations may cause premature neurogenesis in the organoids [71]. One limitation is that fAD accounts for less than 5% of AD cases, and thus, iPSC neuronal models derived from fAD do not represent the majority of cases of AD. Also, fAD-iPSC models primarily accelerate Aβ generation and do not faithfully reflect the gradual, metabolic, and clearance-related failures typical of sAD (Table 1).

### 2.2. Sporadic Disease and Risk Alleles: Tractable Components but Incomplete Phenotype

APOE is the strongest genetic risk factor ever known for AD. APOE has three variants, ApoE2, ApoE3, and ApoE4, of which ApoE4 is strongly associated with sAD, while ApoE2 is known to offer some protection [72,73]. Many studies have demonstrated that APOE4 exacerbates Aβ seeding and suppresses Aβ clearance, thereby increasing AD pathogenesis [74]. Late-onset AD (LOAD) or sAD is heterogeneous, polygenic and age-dependent, which are properties current organoids capture poorly. APOE4 is tractable, but it represents one risk axis rather than the entire sporadic AD spectrum.

Several sAD-specific iPSC models have been generated and studied. iPSC-derived neurons with APOE4 showed an increased number of synapses and increased secretion of Aβ42 compared to cells with APOE3 [57]; however, astrocytes derived from *APOE4* iPSCs showed impaired Aβ uptake and cholesterol accumulation [57]. Because *APOE4*-iPSC-derived microglia also showed a reduced phagocytic capacity for Aβ, it can be deduced that the conversion of *APOE4* to *APOE3* using sAD iPSCs could alleviate multiple AD-related pathologies. Another study demonstrated higher levels of tau phosphorylation and degeneration of GABAergic neurons in *ApoE4*-iPSC neurons [75]. Interestingly, the restoration of these phenotypes by the conversion of *APOE4* to *APOE3* by the gene-editing technology CRISPR/Cas9 system clearly suggests a specific gain-of-toxicity function of *APOE4* in neuronal cells. TREM2, a transmembrane protein, is another sAD risk factor that regulates microglial properties such as phagocytic activities. iPSC microglia derived from sAD patients with an R47H heterozygous TREM2 variant showed deficient signaling with a reduced NLRP3 inflammasome response [76], implying that these iPSC-derived microglia can be used to study pathogenesis and drug screenings.

Organoids have also been successfully derived from AD genetic variants. In a comparative study, organoids derived from *APOE3/3* microglial cells had higher activity of phagocytosis in terms of the ability to internalize Aβ peptides compared to *APOE4/4* [57]. Researchers have also designed several strategies to model sAD organoids, including vascular neuroimmune organoids partly recapitulating the formation of neurovascular unit [77] application of serum to stimulate BBB breakdown [78] as well as inclusion of neurons, astrocytes, microglia and vasculature, and further application of brain extracts from postmortem AD brains to induce pathological seeding [79]. Interestingly, accumulations of phosphorylated tau and Aβ were observed only in iPSC neurons from particular sAD patients and not others [80], suggesting the heterogeneity of sAD patients.

Other groups have reported that these processes are also observed in the sAD organoid model [57,81]. Park et al. reported that plaque-like amyloid aggregates and tau deposition were observed in organoids from sAD and proposed that their highly reproducible human-derived in vitro model is likely to be an effective drug-screening platform [81]. Also, isogenic conversion of APOE4 to APOE3 has been shown to reverse key amyloid, tau, synaptic and neurodegenerative phenotypes, and chimeric organoids that assign distinct neuronal and astrocytic APOE4 have been shown to contribute to tau pathology [82]. This multiple evidence supports the use of iPSC organoids as models of sporadic AD (Table 1). However, one limitation of sAD models is that, due to high genetic variation across different human donors, it is quite difficult to definitively attribute observed neuronal phenotypes strictly to sAD mechanisms rather than simple background genetic noise. Another important methodological limitation is the inability to generate isogenic controls, unlike fAD models, as sAD is polygenic, and creating a true genetically matched healthy control is virtually impossible.

**Table 1:** Alzheimer’s disease (AD): representative iPSC-derived cellular and 3D disease models.

Genetic/Model Context	Model/System	Structural/Genetic Change	Functional/Disease Phenotype	Key Application/Future Implication	Key Reference(s)
APP-E693Δ familial AD	iPSC-derived neurons	APP-E693Δ mutation	Aβ oligomer formation with increased endoplasmic reticulum and oxidative stress; DHA ameliorated the stress phenotype.	Useful for testing approaches that reduce APP-associated amyloid stress and for evaluating candidate neuroprotective compounds such as DHA.	[53]
Engineered familial AD 3D culture	3D human neural culture	Familial AD-associated amyloidogenic context	Extracellular Aβ deposition preceded tau accumulation; γ-secretase inhibition blocked both.	Provides a human 3D platform for testing interventions that alter the relationship between Aβ deposition and downstream tau pathology.	[54]
Human 3D matrix AD model	3D matrix culture	Engineered AD model	Recapitulated amyloid, tau, mitochondrial, and neuronal phenotypes with γ-secretase rescue and microglial uptake.	Allows simultaneous evaluation of multiple AD readouts and therapeutic rescue within one human 3D model.	[55]
Familial AD/Down syndrome	Cerebral organoids	Familial AD mutations or trisomy 21	Amyloid aggregates, tau phosphorylation, and cell death developed without transgene overexpression.	Offers a physiologically relevant system for studying spontaneous amyloid and tau pathology without transgene overexpression.	[66,67]
PITRM1 loss	Cerebral organoids	PITRM1 knockout	Neuronal cell death, tau pathology, and accumulation of Aβ aggregates.	Provides a model for investigating mitochondrial proteostasis as a therapeutic target in AD.	[70]
PSEN1 familial AD	Cerebral organoids	PSEN1 mutation	Premature neurogenesis was observed in organoids carrying some PSEN1 mutations.	Useful for examining how familial AD mutations influence early neural development in addition to later neurodegenerative phenotypes.	[71]
APOE4 sporadic AD risk	iPSC-derived neurons, astrocytes, microglia, and organoids	APOE4 risk allele	Increased neuronal Aβ42 and synaptic changes; impaired astrocytic Aβ uptake and cholesterol handling; reduced microglial phagocytosis; increased tau-related degeneration.	Enables cell-type-specific APOE4 studies and testing of APOE-targeted or gene-correction strategies in patient-relevant human models.	[57,75,82]
TREM2 R47H sporadic AD risk	iPSC-derived microglia	Heterozygous R47H TREM2 variant	Deficient signaling with a reduced NLRP3 inflammasome response.	Provides a human microglial platform for evaluating TREM2-dependent immune dysfunction and potential microglia-directed therapies.	[76]
Sporadic AD patient-derived models	iPSC neurons and organoids	Patient-specific sporadic background; no single causal mutation	Variable Aβ and tau accumulation; plaque-like amyloid and tau in selected organoids; vascular and neuroimmune configurations were used to model sporadic disease.	Supports patient-specific modeling of sporadic AD heterogeneity and evaluation of drug responses across diverse genetic backgrounds.	[77,78,79,80,81]

## 3. Parkinson’s Disease

Parkinson’s disease (PD) is the second most common neurodegenerative disease after AD, with worldwide incidence having doubled over the past 30 years, currently affecting more than 11 million [83]. Like AD, most PD cases are sporadic with unknown etiology, and PD is a multifactorial disease that involves both genetic and environmental factors in its pathogenic mechanism. Less than 15% of cases are monogenic familial PD (fPD) represented by mutations in more than 20 genes [84,85], while the remaining cases are classified as sporadic Parkinson’s disease (sPD) [86,87,88]. The best characterized familial genes are *LRRK2*, which is the most common cause of autosomal dominant disease, and *PRKN*, an autosomal recessive gene, while mutations in genes such as SNCA, PARK2, PINK1, and LRRK2 are closely associated with the onset of both fPD and sPD [89,90,91]. Neuropathologically, PD is characterized by the deposition of α-synuclein that is responsible for Lewy bodies formation, followed by progressive degeneration of DA neurons in the substantia nigra in the midbrain. The clinical manifestations of PD include motor symptoms such as rigidity, tremor, akinesia, and postural instability and nonmotor symptoms including olfactory disturbance, sleep disturbance, and autonomic dysreflexia [92,93,94,95]. Thus, it is believed that the advent of iPSC-derived dopaminergic neurons and organoids would be invaluable not only for elucidating the disease mechanisms but also for screening potential therapeutic agents for PD.

### 3.1. iPSC Dopaminergic Neuronal Models with fPD Mutations

Dopaminergic neurons (DA neurons) derived from iPSCs carrying fPD mutations mimicking disease phenotype, such as selective vulnerability, metabolic abnormalities and neuronal death, have been successfully generated and utilized by many investigators. Those carrying PARK2 and PINK1 [96,97,98,99], LRRK2 [100,101], and SNCA–A53T mutation [102,103] have been demonstrated to exhibit typical PD-like pathological features, including selective accumulation of α-synuclein inclusions, defects in mitochondrial autophagy, increased sensitivity to oxidative stress and elevated ROS levels. Also, iPSC DA neurons derived from patients with triplications or multiplications of the SNCA gene, which encodes α-synuclein protein, have also shown mitochondrial dysfunction, synaptic loss and increased sensitivity to oxidative and endoplasmic reticulum (ER) stress, with a consequent increased neuronal death [104,105,106]. Like neurons, astrocytes derived from iPSCs with LRRK2 mutation [107,108,109] and microglia derived from iPSCs with SNCA and LRRK2 mutations showing several hallmark features of PD have been generated and studied [110,111]. These iPSC-derived neurons, astrocytes and microglia will be excellent tools to further investigate the role of familial genes in PD pathogenesis, which can speed up disease-modifying therapy for PD.

As a further advancement to iPSC-derived DA neurons, 3D organoids have also been successfully developed as vital models to PD mimicking the loss of DA neurons and accumulation of protein aggregates to investigate the pathogenesis of PD. The first midbrain organoid that had the potential to model PD was generated in 2016 [112], and further methodological refining has led to improved organoids that also show the ability to form functional neural networks [113,114]. Specifically, organoids carrying LRRK2 mutations with decreased dendritic complexity and increased DA neuronal death were successfully generated [115], which were partially rescued by inhibiting LRRK2 [116], demonstrating the powerful utility of iPSC-derived organoids to elucidate pathogenesis of PD (Table 2). Similar to AD, one limitation of fPD iPSC neuronal models is that they do not represent the majority of cases of PD. Also, idiopathic PD is caused by a complex interplay between aging, polygenic risk alleles, and environmental factors; however, fPD models cannot inherently capture how these non-genetic factors trigger prompt neurodegeneration.

### 3.2. iPSC Models with sPD Genetic Variants

In addition to fPD models of iPSC neurons, several non-genetic models of iPSCs have been successfully generated and studied as models of sPD from fibroblasts isolated directly from affected patients in the absence of any genetic mutations. Although sPD accounts for most PD cases, they also show α-synuclein accumulation and epigenetic changes in sPD-derived DA neurons similar to the fPD models. Interestingly, some genome-wide association studies (GWAS) have identified some risk factors such as the *GBA* gene, encoding β-glucocerebrosidase as an sPD; however, GBA is also known to be an fPD gene, suggesting that GBA can participate in the pathogenesis of both fPD and sPD [86]. As GBA is a lysosomal encoded gene, the participation of GBA in both fPD and sPD underscores the importance of lysosomal function in both forms of PD. Further, risk variants identified so far for sPD are limited, and they appear to map near fPD genes and lysosome-related genes, but their biological functions are yet to be studied in detail [86]. Other studies have provided evidence that patient-derived sPD models of iPSC neurons show similar pathology and pathways affected as those of fPD neurons, including increased oxidized dopamine levels, mitochondrial defects, lysosomal dysfunction and altered enzyme activities [117,118] as well as changes in microRNAs [101], impaired mitochondrial motility [119] and increased DNA methylation [120].

In addition to iPSC neurons, organoids are also emerging as excellent models to mimic PD. As *GBA1* is the most frequent genetic risk factor for PD, midbrain-like organoids were successfully generated from human embryonic stem cells by deleting GBA1 and overexpressing α-syn [121], and another organoid model with mutant GBA1 was generated that showed the presence of insoluble and S129 phosphorylated α-syn [122] (Table 2). However, it should be noted that these organoid models are short of fibrils, have poor seeding ability and lack typical ultrastructural organization, which are characteristic of patient-derived organoids. In a recent study utilizing an innovative protocol, human iPSC-derived midbrain organoids containing dopaminergic neurons with nigral neuronal identity and characteristics of advanced maturation have also been successfully generated and characterized [123]. These organoids showed insoluble α-synuclein aggregates in the shape of morphologically defined structures mirroring the typical Lewy bodies (LBs) characteristic of PD patient brains. The fact that both ambroxol and GZ667161, the two modulators of the glucocerebrosidase pathway in clinical development, significantly reduced α-synuclein pathology in this iPSC-derived organoid model provided much needed validation for the potential use of these models as sPD organoid models. The fact that LB-like inclusions from these organoids are enriched in crowded organelles and membrane fragments together with the demonstration of comparable seeding activity of the α-syn inclusions clearly indicates that these organoids appropriately recapitulate key aspects of the pathology seen in PD patients. Further development and refinement of PD organoids are necessary, as these models have great potential for disease pathogenesis and drug discovery, which cannot be performed in 2D neuronal models. However, because sPD patients each have completely unique genetic backgrounds, iPSC lines and organoids derived from different sPD patients exhibit immense phenotypic heterogeneity. Additionally, as the process of somatic cell reprogramming wipes away the cell’s epigenetic memory as well as the effects of aging and lifetime environmental toxin exposures, they may not recapitulate the true PD pathology seen in patients.

**Table 2:** Parkinson’s disease (PD): representative iPSC-derived cellular and 3D disease models.

Genetic/Model Context	Model/System	Structural/Genetic Change	Functional/Disease Phenotype	Key Application/Future Implication	Key Reference(s)
PARK2/PINK1 familial PD	iPSC-derived dopaminergic neurons	PARK2 or PINK1 mutation	PINK-1 mutant models showed several PD-associated phenotypes, including α-synuclein accumulation, mitochondrial autophagy defects, oxidative-stress sensitivity, and elevated ROS.	Useful for testing compounds that restore mitochondrial quality control or reduce oxidative stress in mutation-defined dopaminergic neurons.	[96,97,98,99]
LRRK2 familial PD	iPSC-derived dopaminergic neurons	LRRK2 mutation	Disease-associated protein accumulation was accompanied by mitochondrial/autophagic abnormalities, oxidative stress, and neuronal vulnerability.	Provides a human platform for LRRK2 target validation and evaluation of mutation-specific therapeutic rescue.	[100,101]
SNCA-A53T familial PD	iPSC-derived dopaminergic neurons	SNCA-A53T mutation	As part of the familial mutation panel, cells displayed α-synuclein-associated pathology, mitochondrial autophagy defects, oxidative stress, and elevated ROS.	Useful for testing strategies aimed at reducing α-synuclein toxicity in a defined familial PD background.	[102,103]
SNCA gene multiplication	iPSC-derived dopaminergic neurons	SNCA triplication or multiplication	Mitochondrial dysfunction, synaptic loss, increased oxidative and ER stress, and increased neuronal death.	Provides a gene-dosage model for evaluating therapies that reduce α-synuclein burden or protect synaptic and mitochondrial function.	[104,105,106]
LRRK2 astrocytic model	iPSC-derived astrocytes	LRRK2 mutation	α-synuclein accumulation, oxidative stress, reduced viability, and enhanced dopaminergic neuronal death.	Enables study of non-cell-autonomous astrocyte contributions to dopaminergic neuron injury and testing of glia-directed interventions.	[107,108,109]
SNCA/LRRK2 microglial models	iPSC-derived microglia	SNCA or LRRK2 mutation	α-synuclein accumulation, increased inflammation, reduced phagocytic activity, and enhanced dopaminergic neuronal death.	Provides a platform for evaluating microglial inflammatory and phagocytic responses as therapeutic targets in PD.	[110,111]
LRRK2 midbrain organoids	Midbrain organoids	LRRK2 mutation	Decreased dendritic complexity and increased dopaminergic neuronal death; pathology was partially rescued by LRRK2 inhibition.	Offers a 3D system for high-content phenotyping and testing whether LRRK2-directed treatment can rescue disease-relevant neuronal phenotypes.	[115,116]
Sporadic PD patient-derived neurons	iPSC-derived dopaminergic neurons	Sporadic patient background without a defined causal mutation	Increased oxidized dopamine, mitochondrial and lysosomal defects, altered enzyme activities, microRNA changes, impaired mitochondrial motility, and increased DNA methylation.	Supports patient-specific modeling of idiopathic PD and screening for therapies that act on convergent mitochondrial and lysosomal pathways.	[101,117,118,119,120]
GBA1/α-synuclein organoid models	Midbrain-like organoids	GBA1 deletion or mutation, with α-synuclein overexpression in some models	Insoluble and S129-phosphorylated α-synuclein; advanced organoids developed Lewy-body-like inclusions and comparable seeding activity.	Provides a 3D platform for testing glucocerebrosidase-pathway modulators and other strategies that reduce Lewy-body-like α-synuclein pathology.	[121,122,123]

## 4. Amyotrophic Lateral Sclerosis (ALS) and Frontotemporal Dementia (FTD)

ALS and FTD are two ends of the disease spectrum because about 50% of patients with ALS have cognitive impairment to some extent and about 10–15% of ALS patients also reach criteria for diagnosis of FTD [124]. Further, patients with FTD-ALS usually have a shortened life span relative to patients with pure FTD or ALS [125]. ALS is a devastating neurodegenerative disease as the pathology is irreversible and the average time from the onset of symptoms to death or the need for respiratory support is only 3 to 5 years [126,127,128]. As per the recent estimates, more than 40 genes have been linked or associated with ALS [129,130], and mutations in four primary genes, i.e., C9orf72, SOD1, TARDBP, and FUS, account for the majority of fALS cases [129,130]. Most of these fALS genes can be classified based on their function, which falls within the broad categories of protein degradation, trafficking of endolysosomes and autophagy, and protein quality control; however, these classes are not mutually exclusive and overlap to some extent [126,131]. Pathologically, ALS is defined by the progressive degeneration and death of upper and lower motor neurons in the brain and spinal cord, with visible shrinkage and wasting occurring in these regions as the disease progresses [132,133]. The FDA has approved three drugs: the first being Riluzole approved in 1995, the second approval came in 2017 for Edaravone, and the most recent approval in 2023 was for Tofersen that treats ALS linked to a specific mutation in the SOD1 gene [134,135,136]. The available ALS treatments focus on slowing disease progression, managing symptoms, and improving quality of life, as there is currently no cure. Therefore, it is crucial to develop iPSC-derived neuronal and organoid models not only to better understand the disease process but also to speed up the discovery of disease-modifying therapy for ALS-FTD utilizing iPSC neurons and organoids grown from donor somatic cells from ALS-FTD patients.

### 4.1. iPSC Motor Neuronal Models for fALS-FTD

Several iPSC-derived motor neuronal models that recapitulate motor neuronal dysfunction have been generated from ALS-FTD patients with specific mutations that caused the disease, including SOD1, with a variety of mutations [137,138,139,140], TDP43 [141,142,143], C9orf72 [140,144,145,146] and FUS [140]. These iPSC neurons showed dysregulation of neurofilaments, neuronal vulnerability, repeat expansions, RNA toxicity and proteinopathies [137,138,139,140,141,142,143,144,145,146], thus recapitulating motor neuron disease phenotypes and demonstrating usefulness of iPSC-derived neurons as a model of motor neuron dysfunction (Table 3).

Organoids have also been generated starting from patient fibroblasts and then converting into iPSC neurons. iPSC-derived cerebral organoid models from patients with C9-ALS/FTD mutations and C9ORF72-HRE (C9-HRE) carriers as well as controls have been successfully generated and characterized [147]. These organoids exhibited a reduction in the number of deep layer neurons concomitant with molecular, structural, and functional changes in excitatory synapses, thereby validating the model for studies on ALS. Other models of organoids that were developed include one that shows early astrocyte and targeted neuronal pathology with poly-GA dipeptide repeat proteins (DPRs) [148] as well as C9orf72 neuromuscular organoids from ALS patients that manifested the RNA foci and DPRs [149] like ALS-FTD patients. In another study, organoids were generated from iPSCs of ALS-FTD patients harboring the C9orf72- hexanucleotide repeat expansions that demonstrated a higher mtDNA single-nucleotide variant (mtSNV) burden in astroglia when compared to isogenic and healthy controls [150]. These multiple organoid types identify early changes in cell type abundance, developmental processes, and synaptic dysfunction that resonate well with early changes in brain structure and connectivity normally observed in ALS-FTD patients in vivo. However, to see an ALS-like phenotype in a young organoid, it is necessary to expose it to exogenous chemical stressors which introduces experimental artificiality and bypasses the gradual, endogenous decay seen in human patients. Importantly, as organoids lack functional microcirculation, oxygen and nutrients cannot reach the center of the growing tissue, which, over a period of time, can lead to a necrotic core, which kills off cells indiscriminately and introduces confounding cellular stress signals unrelated to fALS-FTD.

### 4.2. iPSC Motor Neuronal Models for sALS-FTD

Like fALS, several iPSC models have been generated to mimic sALS in patients. iPSC-derived motor neurons have been successfully generated from three sALS cases that displayed de novo TDP-43 protein aggregates recapitulating pathology in postmortem brain tissue from one patient from whom the iPSCs were derived [150], thus validating the ALS motor neuron pathology (Table 3). Also, as models of FTD, iPSC neurons were generated from a patient with sporadic FTD and an FTD patient with a novel heterozygous progranulin mutation (PGRN-S116X) [151]. The PGRN levels were reduced in both neurons and microglia, and since the increased sensitivity to kinase inhibitors was rescued by PGRN overexpression, these PGRN-deficient cells may turn out to be compelling iPSC-derived models to study sFTD. Another iPSC-derived neuronal model from an ALS patient carrying the TDP-43-M337V mutation with a two-fold increase in cytosolic TDP-43 was successfully generated to verify whether RNA interference can be used as a therapeutic strategy for sALS-FTD.

Also, an organ-chip model of spinal cord motor neurons derived from iPSCs of a sporadic ALS patient with an integrated blood–brain-like barrier has been generated and characterized [152]. This model has been shown to generate a diversity of mature motor neurons and increased neurofilament levels and may be useful not only to better understand ALS pathology but also for drug screening [152]. However, because organoids derived from iPSCs behave like embryonic or early developmental tissue, they lack the decades of accumulated cellular stress, mitochondrial decline, and aging markers necessary to trigger sporadic disease phenotypes naturally.

**Table 3:** Amyotrophic lateral sclerosis (ALS): representative iPSC-derived cellular and 3D disease models.

Genetic/Model Context	Model/System	Structural/Genetic Change	Functional/Disease Phenotype	Key Application/Future Implication	Key Reference(s)
Familial ALS gene panel	iPSC-derived motor neurons	SOD1, TARDBP/TDP-43, C9orf72, or FUS mutations	Dysregulated neurofilaments, neuronal vulnerability, repeat-expansion/RNA toxicity, and proteinopathies were reproduced across familial ALS models.	Provides mutation-specific human motor-neuron models for target validation and comparison of therapeutic responses across major familial ALS genotypes.	[137,138,139,140,141,142,143,144,145,146]
C9orf72-HRE cerebral organoids	Cerebral organoids	C9orf72 hexanucleotide repeat expansion	Reduced deep-layer neurons with molecular, structural, and functional changes in excitatory synapses.	Useful for studying early cortical and synaptic abnormalities caused by C9orf72 repeat expansion and for testing interventions before overt degeneration.	[147]
C9orf72 poly-GA pathology	Organoids	C9orf72-associated dipeptide repeat pathology	Early astrocyte and targeted neuronal pathology with poly-GA dipeptide repeat proteins.	Provides a multicellular system for evaluating therapies directed at dipeptide-repeat protein toxicity.	[148]
C9orf72 neuromuscular model	Neuromuscular organoids	C9orf72 repeat expansion	RNA foci and dipeptide repeat proteins similar to those observed in ALS-FTD patients.	Extends C9orf72 modeling to the neuromuscular context and can support testing of treatments targeting RNA foci or dipeptide-repeat products.	[149]
C9orf72 astroglial mtDNA phenotype	iPSC-derived organoids	C9orf72 hexanucleotide repeat expansion	Higher mitochondrial DNA single-nucleotide variant burden in astroglia compared with isogenic and healthy controls.	Offers a platform for investigating astroglial mitochondrial abnormalities as a potential therapeutic target in C9orf72-associated disease.	[150]
Sporadic ALS patient model	iPSC-derived motor neurons	Sporadic ALS background	De novo TDP-43 aggregates reproduced pathology seen in postmortem tissue from the corresponding patient.	Supports patient-specific modeling of sporadic ALS and assessment of therapies aimed at TDP-43 aggregation or mislocalization.	[150]
TDP-43-M337V model	iPSC-derived neurons	TDP-43-M337V mutation	Approximately two-fold increase in cytosolic TDP-43; the model was used to test RNA interference as a therapeutic strategy.	Provides a tractable model for evaluating RNA-interference strategies directed at abnormal TDP-43.	[143]
Sporadic ALS spinal-cord organ-chip	Motor-neuron organ-chip with blood–brain-like barrier	Sporadic ALS patient-derived cells	Generated diverse mature motor neurons with increased neurofilament levels.	Adds a blood–brain-like barrier and tissue-interface context that may improve disease modeling and drug-screening relevance for sporadic ALS.	[152]

## 5. Huntington’s Disease

Huntington’s disease (HD) is a fatal neurodegenerative disorder caused by an expanded CAG repeat in the huntingtin gene HTT in an autosomal dominant manner, with longer inherited alleles initiating an earlier age at disease onset [153]. HD affects 6 per 100,000 [154] and is pathologically characterized by progressive motor dysfunction, cognitive impairment, psychiatric abnormalities [155] as well as sleep disturbances [156] and eventually resulting in death [157]. Although many mechanisms have been proposed, the mechanism by which the mutant HTT protein causes neurodegeneration in striatal medium spiny neurons (MSNs) and later to cortical projection neurons is not clearly delineated. In recent years, a major advance has been achieved in the development and characterization of iPSC neurons.

### iPSC Striatal Neuronal Models for HD

The first iPSC-HD neuronal model was generated in 2008 by retroviral transduction of Yamanaka factors, which took about 2 months to complete the reprogramming of human primary fibroblasts starting from biopsy [158]. Following this success, several laboratories generated iPSCs from patients with HD and differentiated them into neurons, such as striatal or cortical neurons, that showed HD-relevant pathology, including changes in mitochondrial function, proteasome activity and autophagy flux [159,160,161,162]. Other investigators used the dual SMAD inhibition method to generate iPSC-derived striatal neurons from patient fibroblasts [163,164]. Further, a total of 14 HD-relevant iPSC lines with varying polyQ-length neurons with polyQ length-dependent pathological features, including decreased bioenergetics, increased lysosomal activity, apoptosis, alterations in cell adhesion, cytoskeleton, and electrophysiology plus decreased survival, have also been developed by HD consortium and other investigators [165,166,167] (Table 4). Importantly, iPSC-derived astrocytes that demonstrated increased vacuoles over time as a non-cell-autonomous model of HD were also successfully generated and characterized [168].

Additionally, development of HD-specific iPSC-derived 3D organoids can also provide new models and new insights into the pathogenesis of HD relevant to in vivo patients. An organoid model was developed by the introduction of 70Q repeats of huntingtin protein, which caused aberrant development of cerebral organoids with loss of neural progenitor organization [169]. As a further advance, in another study, initially organoids from human iPSCs containing functional medium substantia nigra spiny neurons were generated from which assembloids were also successfully generated by assembling striatum-like organoids with substantia nigra-like neurons [170]. These organoids and assembloids could be used for elucidating HD pathogenesis and could be applied as potential drug-screening platforms for HD. Because HD is typically an adult-onset disease, modeling a late-manifesting phenotype in an epigenetically “young” cell is highly challenging. To observe phenotypes like cell death, it is necessary to introduce artificial stressors, such as BDNF withdrawal. Also, a hallmark pathological feature of HD is the formation of intranuclear mutant huntingtin (mHTT) aggregates. However, standard hiPSC-derived striatal neurons often do not exhibit mHTT aggregates in timelines that involve iPSC neuron generation, as aggregate accumulation requires many years in patients. While they might show several functional defects, including metabolic stress or electrophysiological alterations, they fail to spontaneously capture the late-stage protein-aggregation pathology without long-term culture or chemical acceleration. Also, obtaining mature, functional medium spiny neurons (MSNs) primarily destroyed in HD requires complex, lengthy differentiation protocols. These protocols suffer from several limitations, including high line-to-line variability, incomplete or heterogeneous cellular maturation, and low yield of truly mature MSNs compared to other cortical neuron types. Schematics of iPSC neurons and their advantages and limitations are depicted in Figure 2.

**Table 4:** Huntington’s disease (HD): representative iPSC-derived cellular and 3D disease models.

Genetic/Model Context	Model/System	Structural/Genetic Change	Functional/Disease Phenotype	Mechanistic Insight	Key Reference(s)
HTT CAG expansion	Patient-derived HD model context	Expanded CAG repeat in HTT	Longer inherited alleles are associated with earlier disease onset; neurodegeneration prominently affects striatal medium spiny neurons and later cortical projection neurons.	Provides a genetic framework for stratifying HD models by CAG-repeat length and relating repeat burden to disease severity and onset.	[153]
Patient-derived striatal/cortical neurons	iPSC-derived neurons	Mutant HTT/CAG expansion	Changes in mitochondrial function, proteasome activity, and autophagy flux.	Offers patient-derived human neurons for testing therapies that improve mitochondrial, proteostatic, or autophagic dysfunction.	[159,160,161,162]
Dual-SMAD striatal models	iPSC-derived striatal neurons	Patient-derived mutant HTT background	Dual-SMAD inhibition was used to generate disease-relevant striatal neurons from patient fibroblast-derived iPSCs.	Provides a reproducible source of disease-relevant striatal neurons for mechanistic studies and therapeutic screening.	[163,164]
PolyQ-length iPSC panel	Fourteen HD-relevant iPSC lines/neurons	Varying polyQ length	Decreased bioenergetics, increased lysosomal activity and apoptosis, altered cell adhesion, cytoskeleton, and electrophysiology, and decreased survival.	Enables comparison of disease severity across polyQ lengths and identification of phenotypes suitable for quantitative drug-screening readouts.	[165,166,167]
HD astrocytic model	iPSC-derived astrocytes	Mutant HTT background	Increased vacuoles developed over time.	Provides a platform for studying non-cell-autonomous astrocyte contributions and evaluating glial targets in HD.	[168]
70Q cerebral organoid model	Cerebral organoids	70Q huntingtin	Aberrant cerebral organoid development with loss of neural progenitor organization.	Offers a 3D developmental model for testing how mutant huntingtin disrupts neural organization and whether early phenotypes can be rescued.	[169]
Striatum–substantia nigra assembloids	Organoids and assembloids	HD iPSC-derived model	Functional medium spiny neurons were generated, followed by assembly of striatum-like organoids with substantia nigra-like neurons.	Creates a more complex multicellular system for studying striatal interactions and for future circuit-level drug screening.	[170]
CRISPR-corrected HD neurons	HD patient-derived iPSC neurons	CRISPR-Cas9 correction of the HTT mutation	Gene correction reversed the disease phenotype.	Provides an isogenic correction strategy for confirming mutation causality and evaluating gene-editing approaches in patient-derived neurons.	[171]
EVP4593-treated HD neurons	iPSC-induced neurons	Low CAG-repeat expansion in HTT	EVP4593 reduced lysosomes/autophagosomes and store-operated calcium currents and produced neuroprotective effects during cell aging.	Provides a pharmacologic screening model with measurable lysosomal, autophagic, and calcium-signaling readouts for candidate neuroprotective compounds.	[172]

**Figure 2:**
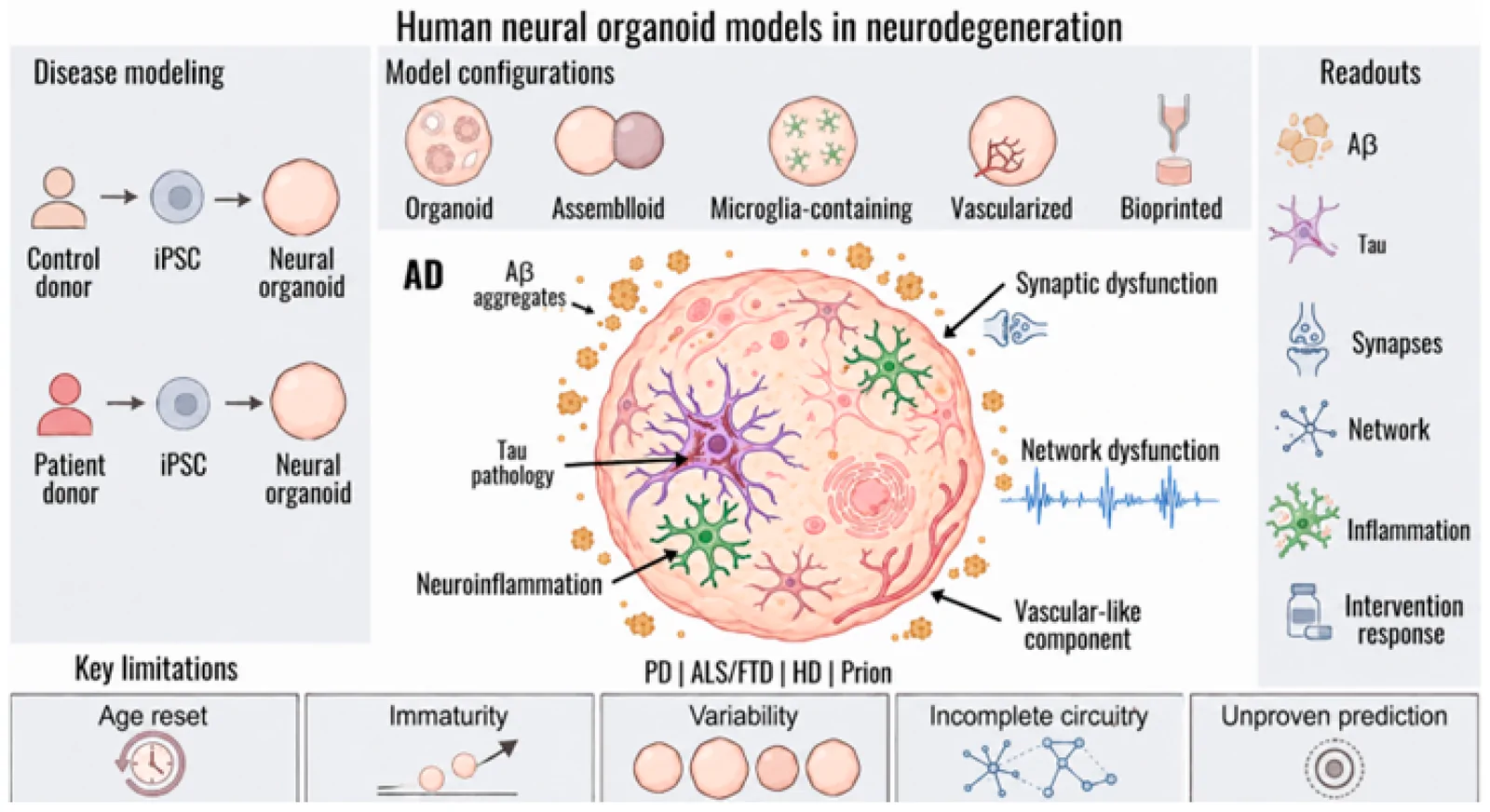
Schematic illustration of generation of patient-derived iPSCs, brain organoids, assembloids and their effect on neural network dysfunction as human cellular models relevant to neurological disorders. The key limitation of this technology is also illustrated.

## 6. Rare Neurological Diseases

In addition to major neurological disorders such as AD, PD, ALS and HD, iPSC-based neuronal models are also commonly used in the study of other rare neurological disorders. Thus, iPSC neurons were successfully generated using somatic cells from patients with spinocerebellar ataxia (SCA1) that showed DNA damage and other pathological features [173,174,175], spinal muscular atrophy (SMA) neurons that showed degenerating neurites and delayed developmental processes [176,177,178], neurons from Rett syndrome (RTT) patients that showed synaptic deficits reflected by fewer glutamatergic synapses, smaller somas, reduced dendritic arborization, and fewer dendritic spines [179,180,181], Fragile X syndrome (FXS) neurons with defects in neurite outgrowth and synaptic dysfunction [182,183,184] and, finally, autism spectrum disorders (ASDs) [185,186,187]. Thus, iPSC-derived neurons have enormous potential for studying rare neurological disorders also.

## 7. The Status of Drug Discovery for Cardinal Neurological Disorders Using iPSC-Derived Cells

While iPSC-derived neurons from somatic cells obtained directly from patients bearing specific disease-associated mutations are crucial for understanding disease pathogenesis, the iPSC neurons are also paramount to screen compounds for identification of novel drug candidates. Using fAD iPSC neurons, 96 hit compounds were identified from the screening of 1684 approved and preclinical drugs that inhibit tau phosphorylation [188]. Among them were four cholesterol lowering statins and another FDA-approved drug efavirenz, an activator of neuron-specific enzyme CYP46A1 [188]. These results support the role of a cholesterol mechanism in the pathogenesis of AD as previously suggested. Another group utilized an FDA-approved drug library of 1258 compounds and screened for Aβ-lowering drugs in iPSC neurons, which resulted in 27 hits; among them, the investigators focused on three drugs, bromocriptine, cromolyn, and topiramate, which together maximized Aβ reduction [189]. These findings even led to the initiation of clinical trials to investigate the safety of bromocriptine [190], and more results are awaited. The other types of compounds that were tested in AD-iPSC cells include gamma-secretase modulators [191,192,193], tau modulators [194], and natural compounds [195]. As currently there is no disease-modifying therapy for AD, the development of iPSC technology is significant for new drug identification.

Similarly, iPSC neurons have a significant role to play in PD therapeutics. Using dopaminergic neurons obtained from familial PD patients with Parkin or PINK1 mutations, four hits, MRS1220, tranylcypromine, flunarizine and bromocriptine, were identified out of 320 compounds by a high-content screening (HCS) assay, which were further tested in idiopathic iPSC-derived neurons [196]. It is important to note that bromocriptine was also a hit drug in another screen using iPSC neurons [189]. In another interesting study, the treatment of midbrain organoids with 2-hydroxypropyl-β-cyclodextrin (2-HP-β-CD), a TFEB and autophagy activator, led to amelioration of the proteomic profile and restoration of levels of TH^+^ dopaminergic neurons [197]. Another autophagy activator BAG956 was also found to be effective in clearance of α-synuclein aggregates in a midbrain organoid model through enhanced autophagy [198]. These examples clearly validate the power of in vitro models in discovering novel drugs.

Several potential drugs have also been identified using ALS-FTD-derived iPSC neurons. By screening more than 6000 compounds utilizing iPSC-derived motor neurons bearing a SOD1 mutation in a cell reporter-based high-throughput screening (HTS), a novel compound was identified that increases NF-L expression by >50% [199], highlighting the utility of iPSC neurons in discovering new compounds for ALS. In another model of iPSC neurons with a C9orf72 mutation, guanosine/cytidine analogs, such as decitabine, entecavir, and nelarabine, were identified as hits out of a total of 1430 compounds screened, and among them, decitabine turned out to be the most potent [200]. Obatoclax was identified as another autophagy activator in a FUS-iPSC-neuronal model that protected neuronal death by reducing FUS protein aggregates [201]. Large-scale drug screening in iPSC-derived motor neurons from sporadic ALS patients also identified baricitinib, memantine and riluzole as a promising therapeutic combination for sALS [202]. It is expected that some of these hit compounds may enter clinical trials in due course, after they are completely vetted for any potential toxicity and bioavailability.

Relative to other neurological disorders, iPSC neurons were less explored towards drug discovery for HD. As the severity of HD is directly influenced by the extent of CAG repeats, CRISPR-Cas9-mediated gene correction was successfully used to reverse the phenotype in the HD patient-derived iPSC neurons [171]. In another study, an iPSC-induced neuronal model was established from patients with low CAG repeat expansion in the HTT gene, and the quinazoline derivative EVP4593 was found to reduce the number of lysosomes/autophagosomes and SOC currents, thereby exerting neuroprotective effects during cell aging [172]. As iPSC-derived neurons are turning out to be more reliable human cellular models, more explorations of this technology together with drug screenings are expected to identify novel drugs for the neurological disorders. The field of drug development holds significant scientific significance in relation to the iPSC-derived neuronal models.

## 8. Cell Therapy for Cardinal Neurological Disorders Using iPSC-Derived Brain Cells

In addition to exploring iPSC-derived neurons in disease pathogenesis and drug discovery, recently, they are also utilized in regenerative medicine through transplantation of neurons to overcome neurodegenerative diseases mainly due to their capacity for self-renewal, differentiation, and modulation of the neural microenvironment. The currently available evidence clearly suggests improved neuron survival, better synaptic structure and function, reduced gliosis and neuroprotection, and some evidence for the restoration of tissue structure. Marked benefits have been reported in rodent or primate models of AD [203], PD [204,205,206], ALS [207,208,209] and HD [210,211,212]. Among the neurological diseases, cell therapy for PD is much advanced relative to other conditions due to long years of experience. Indeed, an open phase I/II study using iPSC-derived DA neurons was initiated at Kyoto University [213]; another study in the US, a phase 1 study, was initiated to test the safety and tolerability of human iPSC-derived dopamine neurons in advanced PD patients [214]; and also, a case of autologous iPSC-derived neuron transplantation was also tried in a single patient [215]. There was also a safety and efficacy study of intrathecal injections of human astrocytes in ALS patients, which demonstrated safety and evidence of a beneficial clinical effect for the first 3 months following cell injection [216]. Also, the first Clinical Trial of Pluripotent Stem Cell-Derived Neural Stem Cells for Huntington’s disease was administered safely in a single patient so far, and more patients are being recruited at UCI, giving much hope to HD patients. A key advantage of iPSCs is their potential for autologous cell therapies that can significantly reduce the risk of immune rejection and associated side effects as the cells are derived from the same patient. If this strategy of personalized medicine is successful, it is likely to offer tremendous benefits to deserving neurological disease patients. Although iPSC-derived neurons are showing promising results, several questions are also raised about safety, stability, and efficacy of iPSCs, which need to be addressed simultaneously. Another important concern is the ability to form tumors, which is a common concern in stem cell research [217]. As described above, several preclinical studies have demonstrated the capacity of iPSC cells to integrate well into host brain circuits, differentiate into many neuronal cell types and glial lineages, and enhance functional recovery. However, many obstacles such as improving cell production, developing distribution systems, and conducting safety evaluations must be addressed to successfully translate stem cell therapies into reality.

## 9. The Advantages and Limitations of 2D Cultures, Organoids, and Organ-on-Chip Models

Although an unprecedented opportunity for simulating human organ functions has been witnessed in recent years using in vitro cellular models such as 2D cultures, organoids, and organ-on-chip, the best model should be selected and utilized for each specific research question as each model has advantages/limitations. While the 2D cultures have advantages of scalability and reproducibility, are cost-effective, and are suitable for early-stage drug screening and basic mechanism studies, the major limitation is the lack of complexity and 3D architecture seen in in vivo organs. Thus, they may not truly represent their intended organ models. Organoids have true 3D tissue complexity and patient-specific genetics suitable for disease modeling and precision medicine; however, they still lack vasculature, which prevents nutrient delivery to the deep core, thereby resulting in necrosis. Because of dynamic fluid flow and precise regulation of the microenvironment, organ-on-chip is particularly useful for pharmacokinetic studies and in multi-organ crosstalk. However, low throughput is the major limitation of this technology. Additionally, these platforms require technical expertise and specialized facilities and additionally involve fabrication complexity. Thus, choosing the right platform depends entirely on the goals of the experiment, the complexity of the disease studied, and the stage of the project.

## 10. Limitations and Prospects

While both iPSC-derived neurons and organoids are explored as potential therapeutic options for cardinal neurological diseases, it is important to note that technology must overcome several limitations before it can be successfully utilized. One major concern is that, because somatic cells are reprogrammed to an embryonic state when converted to iPSC cells, the aging phenotype including epigenetic changes and telomere length will not correspond to the in vivo adult counterparts [218,219]. Also, iPSC-derived neurons often lack full functional and electrophysiological maturity in laboratory dishes. Further, although iPSC-cortical neurons derived from AD patients show increased tau phosphorylation [220], so far, there is no evidence that NFTs are formed in iPSC-derived neurons. Establishing a verified hiPSC line takes 4 to 8 weeks. Once iPSCs are established using transcription factors, three methods are utilized to guide their differentiation into specific, mature cell lineages. These include (1) embryoid body (EB) formation (3D suspension culture), which takes 2–5 days; (2) monolayer directed differentiation (2D culture), which takes a few days to several weeks; and (3) transcription factor-directed forward programming, which takes 4 to 14 days. Additionally, for neuronal cells, network maturity can take anywhere from several weeks to months to form the mature, adult-like synaptic networks that are required for neurotransmission. Importantly, standard 2D cultures fail to replicate the complex 3D tissue architecture and intricate neuro-immune/neuron-glia interactions found in the living human brain [16]. Under these conditions, induced neurons (iNs) generated by directly reprogramming fibroblasts that preserve age-associated epigenetic signatures will be highly useful [38, 221]. Although iPSC organoids are more advanced and can solve some of the issues facing iPSC neurons, they too lack a vascular system and complex cellular interactions, which are vital for long-term survival and to truly reflect the brain function [222]. To be successful, a patient-derived model needs to be generated with all required cell types present at the correct proportions and with the correct cellular organization.

## 11. Commercial Sources of IPSC Cells and Neurons

Axol Biosciences, BitBio, Applied StemCell, Rico Biosciences, FujiFilm Cellular dynamics, BrainXell, Sigma Aldrich, Cyagen, Stem Cell Technologies, iXCells Biotechnologies, Jackson Laboratories, Neuromics, BioRxiv, Abcam, Neuroservices-Alliance, Swiss3RCC, Creative Bioarray.

### 11.1. Sources of Brain Organoids or Media and Supplements

28Bio, OgCelix, Creative Biolabs, StemCell Technologies, ACROBiosystems, NeuraCell.

### 11.2. Sources of Organ-on-a-Chip

Maxwell Biosciences, Corning, Emulate, CN Bio, TissUse GMBH, Mimetas, InSphero, Hesperos Inc., AxoSim, KirkStall Ltd., Nortis.

## 12. Conclusions

Neurological disorders affect over 3.4 billion people globally and 180 million Americans, yet curative or disease-modifying treatments for major conditions like Alzheimer’s, Parkinson’s, and ALS remain vastly insufficient. To address this, induced pluripotent stem cell (iPSC) technology is transforming personalized medicine by converting patient cells into functional human neurons. When combined with CRISPR/Cas9 genome editing, researchers can create precise isogenic controls to accurately evaluate disease pathology and drug responses. Beyond drug discovery, iPSC-derived neurons show immense promise in regenerative medicine via transplantation, with evidence demonstrating improved neuron survival, enhanced synaptic function, and neuroprotection. Ultimately, while modern in vitro systems like 2D cultures, organoids, and organs-on-chips offer unprecedented modeling opportunities, researchers must carefully select the optimal model for their specific experimental questions. Despite remaining obstacles, we are optimistic that iPSC-derived brain cells—including neurons, astrocytes, microglia-like cells, and oligodendrocytes—will ultimately be invaluable for modeling neurological diseases, advancing our understanding of pathogenesis, and driving drug discovery and cell-based therapies.

## Figures and Tables

**Figure 1:**
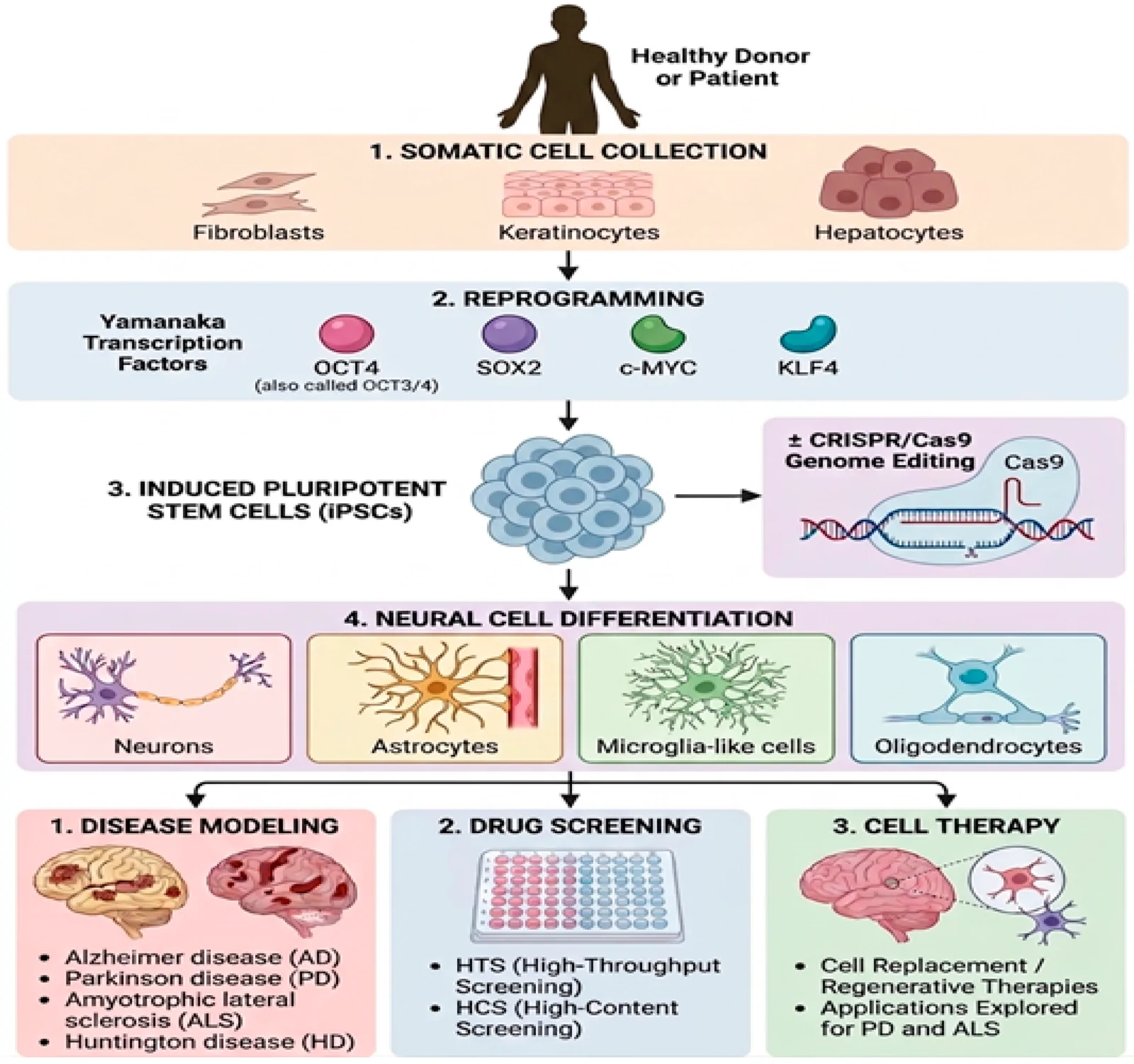
Schematic illustration of reprogramming human somatic cells into iPSCs using Yamanaka factors to generate differentiated brain cell types. CRISPR/Cas9 system can be used to introduce mutations or correction of mutations to generate isogenic control iPSC cells. As indicated, iPSC-derived brain cell types can be used to study disease pathogenesis, drug screening and cell therapy by transplantation.

## Data Availability

No new data were created or analyzed in this study. Data sharing is not applicable to this article.

## References

[1] Joseph A., Nance E. (2022). Nanotherapeutics and the Brain. Annu Rev. Chem. Biomol. Eng..

[2] Gribkoff V.K., Kaczmarek L.K. (2017). The need for new approaches in CNS drug discovery: Why drugs have failed, and what can be done to improve outcomes. Neuropharmacology.

[3] Pankevich D.E., Altevogt B.M., Dunlop J., Gage F.H., Hyman S.E. (2014). Improving and accelerating drug development for nervous system disorders. Neuron.

[4] Ransohoff R.M. (2018). All (animal) models (of neurodegeneration) are wrong. Are they also useful?. J. Exp. Med..

[5] Cummings J. (2018). Lessons Learned from Alzheimer Disease: Clinical Trials with Negative Outcomes. Clin. Transl. Sci..

[6] Hermann D.M., Bacigaluppi M., Bassetti C.L., Bassotti G., Boltze J., Chan A., Dalkara T., Denes A., Diez-Tejedor E., Dodel R. (2025). Most prominent challenges in translational neuroscience and strategic solutions to bridge the gaps: Perspectives from an editorial board interrogation. Explor. Neurosci..

[7] Takahashi K., Yamanaka S. (2006). Induction of pluripotent stem cells from mouse embryonic and adult fibroblast cultures by defined factors. Cell.

[8] Yu J., Vodyanik M.A., Smuga-Otto K., Antosiewicz-Bourget J., Frane J.L., Tian S., Nie J., Jonsdottir G.A., Ruotti V., Stewart R. (2007). Induced pluripotent stem cell lines derived from human somatic cells. Science.

[9] Chun Y.S., Chaudhari P., Jang Y.Y. (2010). Applications of patient-specific induced pluripotent stem cells; focused on disease modeling, drug screening and therapeutic potentials for liver disease. Int. J. Biol. Sci..

[10] Lago S.G., Tomasik J., Bahn S. (2021). Functional patient-derived cellular models for neuropsychiatric drug discovery. Transl. Psychiatry.

[11] Li L., Chao J., Shi Y. (2018). Modeling neurological diseases using iPSC-derived neural cells: iPSC modeling of neurological diseases. Cell Tissue Res..

[12] Lee S., Huang E.J. (2017). Modeling ALS and FTD with iPSC-derived neurons. Brain Res..

[13] Guo X., Wang X., Wang J., Ma M., Ren Q. (2025). Current Development of iPSC-Based Modeling in Neurodegenerative Diseases. Int. J. Mol. Sci..

[14] Kaye J., Reisine T., Finkbeiner S. (2022). Huntington’s disease iPSC models-using human patient cells to understand the pathology caused by expanded CAG repeats. Fac. Rev..

[15] LaMarca E.A., Powell S.K., Akbarian S., Brennand K.J. (2018). Modeling Neuropsychiatric and Neurodegenerative Diseases with Induced Pluripotent Stem Cells. Front. Pediatr..

[16] Wu Y.Y., Chiu F.L., Yeh C.S., Kuo H.C. (2019). Opportunities and challenges for the use of induced pluripotent stem cells in modelling neurodegenerative disease. Open Biol..

[17] Okano H., Morimoto S. (2022). iPSC-based disease modeling and drug discovery in cardinal neurodegenerative disorders. Cell Stem Cell.

[18] Farkhondeh A., Li R., Gorshkov K., Chen K.G., Might M., Rodems S., Lo D.C., Zheng W. (2019). Induced pluripotent stem cells for neural drug discovery. Drug Discov. Today.

[19] Benchoua A., Lasbareilles M., Tournois J. (2021). Contribution of Human Pluripotent Stem Cell-Based Models to Drug Discovery for Neurological Disorders. Cells.

[20] Marei H.E., Khan M.U.A., Hasan A. (2023). Potential use of iPSCs for disease modeling, drug screening, and cell-based therapy for Alzheimer’s disease. Cell. Mol. Biol. Lett..

[21] Song B., Cha Y., Ko S., Jeon J., Lee N., Seo H., Park K.J., Lee I.H., Lopes C., Feitosa M. (2020). Human autologous iPSC-derived dopaminergic progenitors restore motor function in Parkinson’s disease models. J. Clin. Investig..

[22] Rahimi Darehbagh R., Seyedoshohadaei S.A., Ramezani R., Rezaei N. (2024). Stem cell therapies for neurological disorders: Current progress, challenges, and future perspectives. Eur. J. Med. Res..

[23] Zheng Y., Chen X., Bu P., Xue H., Kim D.H., Zhou H., Xia X., Liu Y., Cao Q. (2025). Functional Recovery by Transplantation of Human iPSC-Derived A2B5 Positive Neural Progenitor Cell After Spinal Cord Injury in Mice. Int. J. Mol. Sci..

[24] Kawatani K., Omana Suarez G., Perkerson R.B., Parent E.E., Nambara T., Knight J.A., Parsons T.M., Gupta K., Shue F., Alnobani A. (2025). Human iPSC-Derived MSCs Induce Neurotrophic Effects and Improve Metabolic Activity in Acute Neuronal Injury Models. J. Neurosci..

[25] Ortiz-Virumbrales M., Moreno C.L., Kruglikov I., Marazuela P., Sproul A., Jacob S., Zimmer M., Paull D., Zhang B., Schadt E.E. (2017). CRISPR/Cas9-Correctable mutation-related molecular and physiological phenotypes in iPSC-derived Alzheimer’s PSEN2 ^N141I^ neurons. Acta Neuropathol. Commun..

[26] Franks S.N.J., Heon-Roberts R., Ryan B.J. (2024). CRISPRi: A way to integrate iPSC-derived neuronal models. Biochem Soc. Trans..

[27] Sen T., Thummer R.P. (2022). CRISPR and iPSCs: Recent Developments and Future Perspectives in Neurodegenerative Disease Modelling, Research, and Therapeutics. Neurotox. Res..

[28] McTague A., Rossignoli G., Ferrini A., Barral S., Kurian M.A. (2021). Genome Editing in iPSC-Based Neural Systems: From Disease Models to Future Therapeutic Strategies. Front. Genome Ed..

[29] Pașca S.P. (2018). The rise of three-dimensional human brain cultures. Nature.

[30] Paşca A.M., Sloan S.A., Clarke L.E., Tian Y., Makinson C.D., Huber N., Kim C.H., Park J.Y., O’Rourke N.A., Nguyen K.D. (2015). Functional cortical neurons and astrocytes from human pluripotent stem cells in 3D culture. Nat. Methods.

[31] Castellanos-Montiel M.J., Chaineau M., Franco-Flores A.K., Haghi G., Carrillo-Valenzuela D., Reintsch W.E., Chen C.X., Durcan T.M. (2023). An Optimized Workflow to Generate and Characterize iPSC-Derived Motor Neuron (MN) Spheroids. Cells.

[32] Shima S., Kondo T., Inoue H. (2025). IPSC-derived neural organoids in dementia research: Recent advances and future directions. Neurosci. Res..

[33] Shcheglovitov A., Peterson R.T. (2021). Screening Platforms for Genetic Epilepsies-Zebrafish, iPSC-Derived Neurons, and Organoids. Neurotherapeutics.

[34] Jusop A.S., Thanaskody K., Tye G.J., Dass S.A., Wan Kamarul Zaman W.S., Nordin F. (2023). Development of brain organoid technology derived from iPSC for the neurodegenerative disease modelling: A glance through. Front. Mol. Neurosci..

[35] Arjmand B., KokabiHamidpour S., Rabbani Z., Tayanloo-Beik A., Rahim F., Aghayan H.R., Larijani B. (2022). Organ on a Chip: A Novel in vitro Biomimetic Strategy in Amyotrophic Lateral Sclerosis (ALS) Modeling. Front. Neurol..

[36] Kawakita S., Mandal K., Mou L., Mecwan M.M., Zhu Y., Li S., Sharma S., Hernandez A.L., Nguyen H.T., Maity S. (2022). Organ-On-A-Chip Models of the Blood-Brain Barrier: Recent Advances and Future Prospects. Small.

[37] Ferrari I., Limiti E., Giannitelli S.M., Trombetta M., Rainer A., D’Amelio M., La Barbera L., Gori M. (2025). Microfluidic-Based Technologies for Crossing the Blood-Brain Barrier Against Alzheimer’s Disease: Novel Strategies and Challenges. Int. J. Mol. Sci..

[38] Vierbuchen T., Ostermeier A., Pang Z.P., Kokubu Y., Südhof T.C., Wernig M. (2010). Direct conversion of fibroblasts to functional neurons by defined factors. Nature.

[39] Mertens J., Paquola A.C.M., Ku M., Hatch E., Böhnke L., Ladjevardi S., McGrath S., Campbell B., Lee H., Herdy J.R. (2015). Directly Reprogrammed Human Neurons Retain Aging-Associated Transcriptomic Signatures and Reveal Age-Related Nucleocytoplasmic Defects. Cell Stem Cell.

[40] Richner M., Victor M.B., Liu Y., Abernathy D., Yoo A.S. (2015). MicroRNA-based conversion of human fibroblasts into striatal medium spiny neurons. Nat. Protoc..

[41] Church V.A., Cates K., Capano L., Aryal S., Kim W.K., Yoo A.S. (2021). Generation of Human Neurons by microRNA-Mediated Direct Conversion of Dermal Fibroblasts. Methods Mol. Biol..

[42] Breijyeh Z., Karaman R. (2020). Comprehensive Review on Alzheimer’s Disease: Causes and Treatment. Molecules.

[43] Soria Lopez J.A., González H.M., Léger G.C. (2019). Alzheimer’s disease. Handbook of Clinical Neurology.

[44] Atri A. (2019). The Alzheimer’s Disease Clinical Spectrum: Diagnosis and Management. Med. Clin. N. Am..

[45] Li X., Feng X., Sun X., Hou N., Han F., Liu Y. (2022). Global, regional, and national burden of Alzheimer’s disease and other dementias, 1990-2019. Front. Aging Neurosci..

[46] Duan Y., Han C., Zheng H., Yu J., Luo M. (2025). Global, regional, and national burden of Alzheimer’s disease and other dementias from 1990 to 2021: Findings from the Global Burden of Disease Study 2021. Front. Aging Neurosci..

[47] Yagi T., Ito D., Okada Y., Akamatsu W., Nihei Y., Yoshizaki T., Yamanaka S., Okano H., Suzuki N. (2011). Modeling familial Alzheimer’s disease with induced pluripotent stem cells. Hum. Mol. Genet..

[48] Cacace R., Sleegers K., Van Broeckhoven C. (2016). Molecular genetics of early-onset Alzheimer’s disease revisited. Alzheimers Dement.

[49] Dorszewska J., Prendecki M., Oczkowska A., Dezor M., Kozubski W. (2016). Molecular Basis of Familial and Sporadic Alzheimer’s Disease. Curr. Alzheimer Res..

[50] EADB, EADI, Bonn, ADGC, CHARGE, FinnGen, GERAD, GR@ACE/DEGESCO, PGC-ALZ (2026). Consensus meta-analysis of genome-wide association studies for Alzheimer’s disease and related dementias. Nat. Genet..

[51] Ochalek A., Mihalik B., Avci H.X., Chandrasekaran A., Téglási A., Bock I., Giudice M.L., Táncos Z., Molnár K., László L. (2017). Neurons derived from sporadic Alzheimer’s disease iPSCs reveal elevated TAU hyperphosphorylation, increased amyloid levels, and GSK3B activation. Alzheimers Res. Ther..

[52] Verheijen M.C.T., Krauskopf J., Caiment F., Nazaruk M., Wen Q.F., van Herwijnen M.H.M., Hauser D.A., Gajjar M., Verfaillie C., Vermeiren Y. (2022). IPSC-derived cortical neurons to study sporadic Alzheimer disease: A transcriptome comparison with post-mortem brain samples. Toxicol. Lett..

[53] Kondo T., Asai M., Tsukita K., Kutoku Y., Ohsawa Y., Sunada Y., Imamura K., Egawa N., Yahata N., Okita K. (2013). Modeling Alzheimer’s disease with iPSCs reveals stress phenotypes associated with intracellular Aβ and differential drug responsiveness. Cell Stem Cell.

[54] Choi S.H., Kim Y.H., Hebisch M., Sliwinski C., Lee S., D’avanzo C., Chen H., Hooli B., Asselin C., Muffat J. (2014). A three-dimensional human neural cell culture model of Alzheimer’s disease. Nature.

[55] Hebisch M., Kamin V., Cenini G., Piazzesi A., Bertan F., Weykopf B., Schlee J., Kaniyappan S., Washicosky K.J., Kim D.Y. (2026). Recapitulation of plaque formation, tau pathology, and neurodegeneration in a human 3D matrix model of Alzheimer’s disease. Cell Rep. Methods.

[56] Abud E.M., Ramirez R.N., Martinez E.S., Healy L.M., Nguyen C.H.H., Newman S.A., Yeromin A.V., Scarfone V.M., Marsh S.E., Fimbres C. (2017). IPSC-Derived Human Microglia-like Cells to Study Neurological Diseases. Neuron.

[57] Lin Y.T., Seo J., Gao F., Feldman H.M., Wen H.L., Penney J., Cam H.P., Gjoneska E., Raja W.K., Cheng J. (2018). APOE4 Causes Widespread Molecular and Cellular Alterations Associated with Alzheimer’s Disease Phenotypes in Human iPSC-Derived Brain Cell Types. Neuron.

[58] Claes C., Van Den Daele J., Boon R., Schouteden S., Colombo A., Monasor L.S., Fiers M., Ordovás L., Nami F., Bohrmann B. (2019). Human stem cell-derived monocytes and microglia-like cells reveal impaired amyloid plaque clearance upon heterozygous or homozygous loss of TREM2. Alzheimers Dement.

[59] Oksanen M., Petersen A.J., Naumenko N., Puttonen K., Lehtonen Š., Olivé M.G., Shakirzyanova A., Leskelä S., Sarajärvi T., Viitanen M. (2017). PSEN1 Mutant iPSC-Derived Model Reveals Severe Astrocyte Pathology in Alzheimer’s Disease. Stem Cell Rep..

[60] Jones V.C., Atkinson-Dell R., Verkhratsky A., Mohamet L. (2017). Aberrant iPSC-derived human astrocytes in Alzheimer’s disease. Cell Death Dis..

[61] Rowland H.A., Miller G., Liu Q., Li S., Sharp N.R., Ng B., Wei T., Arunasalam K., Koychev I., Hedegaard A. (2025). Changes in iPSC-astrocyte morphology reflect Alzheimer’s disease patient clinical markers. Stem Cells.

[62] Wang S., Bates J., Li X., Schanz S., Chandler-Militello D., Levine C., Maherali N., Studer L., Hochedlinger K., Windrem M. (2013). Human iPSC-derived oligodendrocyte progenitor cells can myelinate and rescue a mouse model of congenital hypomyelination. Cell Stem Cell.

[63] Hu B.Y., Du Z.W., Zhang S.C. (2009). Differentiation of human oligodendrocytes from pluripotent stem cells. Nat. Protoc..

[64] Douvaras P., Fossati V. (2015). Generation and isolation of oligodendrocyte progenitor cells from human pluripotent stem cells. Nat. Protoc..

[65] Park J., Kang S., Kim S., Kim D., Shin B., Mun J.Y., Park Y., Kim J., Goldman S.A., Kim K.P. (2026). Generation of Functional Oligodendrocyte Progenitor Cells Through Serial Replating of iPSC-Derived NPC Spheres. Cells.

[66] Raja W.K., Mungenast A.E., Lin Y.T., Ko T., Abdurrob F., Seo J., Tsai L.H. (2016). Self-Organizing 3D Human Neural Tissue Derived from Induced Pluripotent Stem Cells Recapitulate Alzheimer’s Disease Phenotypes. PLoS ONE.

[67] Gonzalez C., Armijo E., Bravo-Alegria J., Becerra-Calixto A., Mays C.E., Soto C. (2018). Modeling amyloid beta and tau pathology in human cerebral organoids. Mol. Psychiatry.

[68] Dooling B.R., Vielle A., Lucero E.M., Rydland C., Quang D., Summers R., Esquer H., Coughlan C., Galbraith M.D., Espinosa J.M. (2026). Trisomy 21 cerebral organoids exhibit Alzheimer’s disease amyloid and apolipoprotein E co-pathologies. bioRxiv.

[69] Fertan E., Böken D., Murray A., Danial J.S.H., Lam J.Y.L., Wu Y., Goh P.A., Alić I., Cheetham M.R., Lobanova E. (2024). Cerebral organoids with chromosome 21 trisomy secrete Alzheimer’s disease-related soluble aggregates detectable by single-molecule-fluorescence and super-resolution microscopy. Mol. Psychiatry.

[70] Pérez M.J., Ivanyuk D., Panagiotakopoulou V., Di Napoli G., Kalb S., Brunetti D., Al-Shaana R., Kaeser S.A., Fraschka S.A., Jucker M. (2021). Loss of function of the mitochondrial peptidase PITRM1 induces proteotoxic stress and Alzheimer’s disease-like pathology in human cerebral organoids. Mol. Psychiatry.

[71] Arber C., Lovejoy C., Harris L., Willumsen N., Alatza A., Casey J.M., Lines G., Kerins C., Mueller A.K., Zetterberg H. (2021). Familial Alzheimer’s Disease Mutations in PSEN1 Lead to Premature Human Stem Cell Neurogenesis. Cell Rep..

[72] Corder E.H., Saunders A.M., Strittmatter W.J., Schmechel D.E., Gaskell P.C., Small G.W., Roses A.D., Haines J.L., Pericak-Vance M.A. (1993). Gene dose of apolipoprotein E type 4 allele and the risk of Alzheimer’s disease in late onset families. Science.

[73] Kim J., Basak J.M., Holtzman D.M. (2009). The role of apolipoprotein E in Alzheimer’s disease. Neuron.

[74] Long J.M., Holtzman D.M. (2019). Alzheimer Disease: An Update on Pathobiology and Treatment Strategies. Cell.

[75] Wang C., Najm R., Xu Q., Jeong D.E., Walker D., Balestra M.E., Yoon S.Y., Yuan H., Li G., Miller Z.A. (2018). Gain of toxic apolipoprotein E4 effects in human iPSC-derived neurons is ameliorated by a small-molecule structure corrector. Nat. Med..

[76] Cosker K., Mallach A., Limaye J., Piers T.M., Staddon J., Neame S.J., Hardy J., Pocock J.M. (2021). Microglialsignalling pathway deficits associated with the patient derived R47H TREM2 variants linked to AD indicate inability to activate inflammasome. Sci. Rep..

[77] Kistemaker L., van Bodegraven E.J., de Vries H.E., Hol E.M. (2025). Vascularized human brain organoids: Current possibilities and prospects. Trends Biotechnol..

[78] Chen X., Sun G., Tian E., Zhang M., Davtyan H., Beach T.G., Reiman E.M., Blurton-Jones M., Holtzman D.M., Shi Y. (2021). Modeling Sporadic Alzheimer’s Disease in Human Brain Organoids under Serum Exposure. Adv. Sci..

[79] Ji Y., Chen X., Wang Z., Meek C.J., McLean J.L., Yang Y., Yuan C., Rochet J.C., Liu F., Xu R. (2025). Alzheimer’s disease patient brain extracts induce multiple pathologies in novel vascularized neuroimmune organoids for disease modeling and drug discovery. Mol. Psychiatry.

[80] Israel M.A., Yuan S.H., Bardy C., Reyna S.M., Mu Y., Herrera C., Hefferan M.P., Van Gorp S., Nazor K.L., Boscolo F.S. (2012). Probing sporadic and familial Alzheimer’s disease using induced pluripotent stem cells. Nature.

[81] Park J.C., Jang S.Y., Lee D., Lee J., Kang U., Chang H., Kim H.J., Han S.H., Seo J., Choi M. (2021). A logical network-based drug-screening platform for Alzheimer’s disease representing pathological features of human brain organoids. Nat. Commun..

[82] Huang S., Zhang Z., Cao J., Yu Y., Pei G. (2022). Chimeric cerebral organoids reveal the essentials of neuronal and astrocytic APOE4 for Alzheimer’s tau pathology. Signal Transduct. Target. Ther..

[83] Li M., Ye X., Huang Z., Ye L., Chen C. (2025). Global burden of Parkinson’s disease from 1990 to 2021: A population-based study. BMJ Open.

[84] Tran J., Anastacio H., Bardy C. (2020). Genetic predispositions of Parkinson’s disease revealed in patient-derived brain cells. npj Park. Dis..

[85] Di Fonzo A., Percetti M., Monfrini E. (2023). Harmonizing genetic testing for Parkinson’s disease: Results of the PARKNET multicentric study. Mov. Disord..

[86] Blauwendraat C., Nalls M.A., Singleton A.B. (2020). The genetic architecture of Parkinson’s disease. Lancet Neurol..

[87] Imaizumi Y., Okano H. (2014). Modeling human neurological disorders with induced pluripotent stem cells. J. Neurochem..

[88] Vázquez-Vélez G.E., Zoghbi H.Y. (2021). Parkinson’s Disease Genetics and Pathophysiology. Annu Rev. Neurosci..

[89] Lunati A., Lesage S., Brice A. (2018). The genetic landscape of Parkinson’s disease. Rev. Neurol..

[90] Jia F., Fellner A., Kumar K.R. (2022). Monogenic Parkinson’s disease: Genotype, phenotype, pathophysiology, and genetic testing. Genes.

[91] Cook L., Schulze J., Verbrugge J. (2021). The commercial genetic testing landscape for Parkinson’s disease. Park. Relat. Disord..

[92] Corti O., Lesage S., Brice A. (2011). What genetics tells us about the causes and mechanisms of Parkinson’s disease. Physiol. Rev..

[93] Poewe W. (2008). Non-motor symptoms in Parkinson’s disease. Eur. J. Neurol..

[94] Pfeiffer R.F. (2016). Non-motor symptoms in Parkinson’s disease. Park. Relat. Disord..

[95] Kwon K.Y., Kang S.H., Kim M., Lee H.M., Jang J.W., Kim J.Y., Lee S.M., Koh S.B. (2014). Nonmotor Symptoms and Cognitive Decline in de novo Parkinson’s Disease. Can. J. Neurol. Sci..

[96] Jiang H., Ren Y., Yuen E.Y., Zhong P., Ghaedi M., Hu Z., Azabdaftari G., Nakaso K., Yan Z., Feng J. (2012). Parkin controls dopamine utilization in human midbrain dopaminergic neurons derived from induced pluripotent stem cells. Nat. Commun..

[97] Imaizumi Y., Okada Y., Akamatsu W., Koike M., Kuzumaki N., Hayakawa H., Nihira T., Kobayashi T., Ohyama M., Sato S. (2012). Mitochondrial dysfunction associated with increased oxidative stress and alpha-synuclein accumulation in PARK2 iPSC-derived neurons and postmortem brain tissue. Mol. Brain..

[98] Chung S.Y., Kishinevsky S., Mazzulli J.R., Graziotto J., Mrejeru A., Mosharov E.V., Puspita L., Valiulahi P., Sulzer D., Milner T.A. (2016). Parkin and PINK1 Patient iPSC-Derived Midbrain Dopamine Neurons Exhibit Mitochondrial Dysfunction and alpha-Synuclein Accumulation. Stem Cell Rep..

[99] Oh C.K., Sultan A., Platzer J., Dolatabadi N., Soldner F., McClatchy D.B., Diedrich J.K., Yates J.R., Ambasudhan R., Nakamura T. (2017). S-Nitrosylation of PINK1 Attenuates PINK1/Parkin-Dependent Mitophagy in hiPSC-Based Parkinson’s Disease Models. Cell Rep..

[100] Liu G.H., Qu J., Suzuki K., Nivet E., Li M., Montserrat N., Yi F., Xu X., Ruiz S., Zhang W. (2012). Progressive degeneration of human neural stem cells caused by pathogenic LRRK2. Nature.

[101] Tolosa E., Botta-Orfila T., Morato X., Calatayud C., Ferrer-Lorente R., Marti M.J., Fernández M., Gaig C., Raya A., Consiglio A. (2018). MicroRNA alterations in iPSC-derived dopaminergic neurons from Parkinson disease patients. Neurobiol. Aging..

[102] Kouroupi G., Taoufik E., Vlachos I.S., Tsioras K., Antoniou N., Papastefanaki F., Chroni-Tzartou D., Wrasidlo W., Bohl D., Stellas D. (2017). Defective synaptic connectivity and axonal neuropathology in a human iPSC-based model of familial Parkinson’s disease. Proc. Natl. Acad. Sci. USA.

[103] Ryan S.D., Dolatabadi N., Chan S.F., Zhang X., Akhtar M.W., Parker J., Soldner F., Sunico C.R., Nagar S., Talantova M. (2013). Isogenic human iPSC Parkinson’s model shows nitrosative stress-induced dysfunction in MEF2-PGC1 transcription. Cell.

[104] Zambon F., Cherubini M., Fernandes H.J.R., Lang C., Ryan B.J., Volpato V., Bengoa-Vergniory N., Vingill S., Attar M., Booth H.D.E. (2019). Cellular alpha-synuclein pathology is associated with bioenergetic dysfunction in Parkinson’s iPSC-derived dopamine neurons. Hum. Mol. Genet..

[105] Chung C.Y., Khurana V., Auluck P.K., Tardiff D.F., Mazzulli J.R., Soldner F., Baru V., Lou Y., Freyzon Y., Cho S. (2013). Identification and rescue of alpha-synuclein toxicity in Parkinson patient-derived neurons. Science.

[106] Ludtmann M.H.R., Angelova P.R., Horrocks M.H., Choi M.L., Rodrigues M., Baev A.Y., Berezhnov A.V., Yao Z., Little D., Banushi B. (2018). Alpha-synuclein oligomers interact with ATP synthase and open the permeability transition pore in Parkinson’s disease. Nat. Commun..

[107] Angelique di Domenico A., Carola G., Calatayud C., Pons-Espinal M., Muñoz J.P., Richaud-Patin Y., Fernandez-Carasa I., Gut M., Faella A., Parameswaran J. (2019). Patient-specific iPSC-Derived astrocytes contribute to non-cell-autonomous neurodegeneration in Parkinson’s disease. Stem Cell Rep..

[108] Ibarra-Aizpurua N., Olano-Bringas J., Vallin B., Crompton L.A., Cowley S.A., Bengoa-Vergniory N., Wade-Martins R. (2026). Reactive astrocytes mediate toxicity in iPSC derived dopaminergic neurons. npj Park. Dis..

[109] Ramos-Gonzalez P., Mato S., Chara J.C., Verkhratsky A., Matute C., Cavaliere F. (2021). Astrocytic atrophy as a pathological feature of Parkinson’s disease with LRRK2 mutation. npj Park. Dis..

[110] Badanjak K., Mulica P., Smajic S., Delcambre S., Tranchevent L.C., Diederich N., Rauen T., Schwamborn J.C., Glaab E., Cowley S.A. (2021). IPSC-Derived Microglia as a Model to Study Inflammation in Idiopathic Parkinson’s Disease. Front. Cell Dev. Biol..

[111] Ohtonen S., Giudice L., Jäntti H., Fazaludeen M.F., Shakirzyanova A., Gómez-Budia M., Välimäki N.N., Niskanen J., Korvenlaita N., Fagerlund I. (2023). Human iPSC-derived microglia carrying the LRRK2-G2019S mutation show a Parkinson’s disease related transcriptional profile and function. Sci. Rep..

[112] Jo J., Xiao Y., Sun A.X., Cukuroglu E., Tran H.-D., Göke J., Tan Z.Y., Saw T.Y., Tan C.-P., Lokman H. (2016). Midbrain-like Organoids from Human Pluripotent Stem Cells Contain Functional Dopaminergic and Neuromelanin-Producing Neurons. Cell Stem Cell.

[113] Kelava I., Lancaster M.A. (2016). Dishing out mini-brains: Current progress and future prospects in brain organoid research. Dev. Biol..

[114] Monzel A.S., Smits L.M., Hemmer K., Hachi S., Moreno E.L., van Wuellen T., Jarazo J., Walter J., Brüggemann I., Boussaad I. (2017). Derivation of Human Midbrain-Specific Organoids from Neuroepithelial Stem Cells. Stem Cell Rep..

[115] Smits L.M., Reinhardt L., Reinhardt P., Glatza M., Monzel A.S., Stanslowsky N., Rosato-Siri M.D., Zanon A., Antony P.M., Bellmann J. (2019). Modeling Parkinson’s disease in midbrain-like organoids. npj Park. Dis..

[116] Bolognin S., Fossepre M., Qing X., Jarazo J., Scancar J., Moreno E.L., Nickels S.L., Wasner K., Ouzren N., Walter J. (2019). 3D Cultures of Parkinson’s Disease-Specific Dopaminergic Neurons for High Content Phenotyping and Drug Testing. Adv. Sci..

[117] Burbulla L.F., Song P., Mazzulli J.R., Zampese E., Wong Y.C., Jeon S., Santos D.P., Blanz J., Obermaier C.D., Strojny C. (2017). Dopamine oxidation mediates mitochondrial and lysosomal dysfunction in Parkinson’s disease. Science.

[118] Mazzulli J.R., Zunke F., Tsunemi T., Toker N.J., Jeon S., Burbulla L.F., Patnaik S., Sidransky E., Marugan J.J., Sue C.M. (2016). Activation of beta-Glucocerebrosidase Reduces Pathological alpha-Synuclein and Restores Lysosomal Function in Parkinson’s Patient Midbrain Neurons. J. Neurosci..

[119] Hsieh C.-H., Shaltouki A., Gonzalez A.E., da Cruz A.B., Burbulla L.F., Lawrence E.S., Schüle B., Krainc D., Palmer T.D., Wang X. (2016). Functional Impairment in Miro Degradation and Mitophagy Is a Shared Feature in Familial and Sporadic Parkinson’s Disease. Cell Stem Cell.

[120] Fernández-Santiago R., Merkel A., Castellano G., Heath S., Raya Á., Tolosa E., Martí M.-J., Consiglio A., Ezquerra M. (2019). Whole-genome DNA hyper-methylation in iPSC-derived dopaminergic neurons from Parkinson’s disease patients. Clin. Epigenet..

[121] Jo J., Yang L., Tran H., Yu W., Sun A.X., Chang Y.Y., Jung B.C., Lee S., Saw T.Y., Xiao B. (2021). Lewy body-like inclusions in human midbrain organoids carrying glucocerebrosidase and α-synuclein mutations. Ann. Neurol..

[122] Baden P., Perez M.J., Raji H., Bertoli F., Kalb S., Illescas M., Spanos F., Giuliano C., Calogero A.M., Oldrati M. (2023). Glucocerebrosidase is imported into mitochondria and preserves complex I integrity and energy metabolism. Nat. Commun..

[123] Frattini E., Faustini G., Lopez G., Carsana E.V., Tosi M., Trezzi I., Magni M., Soldà G., Straniero L., Facchi D. (2025). Lewy pathology formation in patient-derived GBA1 Parkinson’s disease midbrain organoids. Brain.

[124] Goldstein L.H., Abrahams S. (2013). Changes in cognition and behaviour in amyotrophic lateral sclerosis: Nature of impairment and implications for assessment. Lancet Neurol..

[125] Olney R.K., Murphy J., Forshew D., Garwood E., Miller B.L., Langmore S., Kohn M.A., Lomen-Hoerth C. (2005). The effects of executive and behavioral dysfunction on the course of ALS. Neurology.

[126] Brown R.H., Al-Chalabi A. (2017). Amyotrophic Lateral Sclerosis. N. Engl. J. Med..

[127] Okano H., Yasuda D., Fujimori K., Morimoto S., Takahashi S. (2020). Ropinirole, a New ALS Drug Candidate Developed Using iPSCs. Trends Pharmacol. Sci..

[128] Talbott E.O., Malek A.M., Lacomis D. (2016). The epidemiology of amyotrophic lateral sclerosis. Handb. Clin. Neurol..

[129] Nijs M., Van Damme P. (2024). The genetics of amyotrophic lateral sclerosis. Curr. Opin. Neurol..

[130] Wang H., Guan L., Deng M. (2023). Recent progress of the genetics of amyotrophic lateral sclerosis and challenges of gene therapy. Front. Neurosci..

[131] Taylor J.P., Brown R.H., Cleveland D.W. (2016). Decoding ALS: From genes to mechanism. Nature.

[132] Saberi S., Stauffer J.E., Schulte D.J., Ravits J. (2015). Neuropathology of Amyotrophic Lateral Sclerosis and Its Variants. Neurol. Clin..

[133] Yoshida M. (2019). Neuropathology of Amyotrophic Lateral Sclerosis. Brain Nerve.

[134] Miller R.G., Mitchell J.D., Moore D.H. (2012). Riluzole for amyotrophic lateral sclerosis (ALS)/motor neuron disease (MND). Cochrane Database Syst. Rev..

[135] Neupane P., Thada P.K., Singh P., Faisal A.R., Rai N., Poudel P., Waleed M.S., Quinonez J., Ruxmohan S., Jain E. (2023). Investigating Edaravone Use for Management of Amyotrophic Lateral Sclerosis (ALS): A Narrative Review. Cureus.

[136] Everett W.H., Bucelli R.C. (2024). Tofersen for SOD1 ALS. Neurodegener. Dis. Manag.

[137] Chen H., Qian K., Du Z., Cao J., Petersen A., Liu H., Blackbourn L.W., Huang C.-L., Errigo A., Yin Y. (2014). Modeling ALS with iPSCs reveals that mutant SOD1 misregulates neurofilament balance in motor neurons. Cell Stem Cell.

[138] Kiskinis E., Sandoe J., Williams L.A., Boulting G.L., Moccia R., Wainger B.J., Han S., Peng T., Thams S., Mikkilineni S. (2014). Pathways disrupted in human ALS motor neurons identified through genetic correction of mutant SOD1. Cell Stem Cell.

[139] Yang Y.M., Gupta S.K., Kim K.J., Powers B.E., Cerqueira A., Wainger B.J., Ngo H.D., Rosowski K.A., Schein P.A., Ackeifi C.A. (2013). A small molecule screen in stem-cell-derived motor neurons identifies a kinase inhibitor as a candidate therapeutic for ALS. Cell Stem Cell.

[140] Wainger B.J., Kiskinis E., Mellin C., Wiskow O., Han S.S., Sandoe J., Perez N.P., Williams L.A., Lee S., Boulting G. (2014). Intrinsic membrane hyperexcitability of amyotrophic lateral sclerosis patient-derived motor neurons. Cell Rep..

[141] Bilican B., Serio A., Barmada S.J., Nishimura A.L., Sullivan G.J., Carrasco M., Phatnani H.P., Puddifoot C.A., Story D., Fletcher J. (2012). Mutant induced pluripotent stem cell lines recapitulate aspects of TDP-43 proteinopathies and reveal cell-specific vulnerability. Proc. Natl. Acad. Sci. USA.

[142] Serio A., Bilican B., Barmada S.J., Ando D.M., Zhao C., Siller R., Burr K., Haghi G., Story D., Nishimura A.L. (2013). Astrocyte pathology and the absence of non-cell autonomy in an induced pluripotent stem cell model of TDP-43 proteinopathy. Proc. Natl. Acad. Sci. USA.

[143] Nishimura A.L., Shum C., Scotter E.L., Abdelgany A., Sardone V., Wright J., Lee Y.-B., Chen H.-J., Bilican B., Carrasco M. (2014). Allele-specific knockdown of ALS-associated mutant TDP-43 in neural stem cells derived from induced pluripotent stem cells. PLoS ONE.

[144] Donnelly C.J., Zhang P.W., Pham J.T., Haeusler A.R., Mistry N.A., Vidensky S., Daley E.L., Poth E.M., Hoover B., Fines D.M. (2013). RNA toxicity from the ALS/FTD C9ORF72 expansion is mitigated by antisense intervention. Neuron.

[145] Sareen D., O’Rourke J.G., Meera P., Muhammad A.K., Grant S., Simpkinson M., Bell S., Carmona S., Ornelas L., Sahabian A. (2013). Targeting RNA foci in iPSC-derived motor neurons from ALS patients with a C9ORF72 repeat expansion. Sci. Transl. Med..

[146] Burley S., Beccano-Kelly D.A., Talbot K., Llana O.C., Wade-Martins R. (2022). Hyperexcitability in young iPSC-derived C9ORF72 mutant motor neurons is associated with increased intracellular calcium release. Sci. Rep..

[147] van der Geest A.T., Jakobs C.E., Ljubikj T., Huffels C.F.M., Cañizares Luna M., Vieira de Sá R., Adolfs Y., de Wit M., Rutten D.H., Kaal M. (2024). Molecular pathology, developmental changes and synaptic dysfunction in (pre-) symptomatic human C9ORF72-ALS/FTD cerebral organoids. Acta Neuropathol. Commun..

[148] Szebényi K., Wenger L.M.D., Sun Y., Dunn A.W.E., Limegrover C.A., Gibbons G.M., Conci E., Paulsen O., Mierau S.B., Balmus G. (2021). Human ALS/FTD brain organoid slice cultures display distinct early astrocyte and targetable neuronal pathology. Nat. Neurosci..

[149] Gao C., Shi Q., Pan X., Chen J., Zhang Y., Lang J., Wen S., Liu X., Cheng T.-L., Lei K. (2024). Neuromuscular organoids model spinal neuromuscular pathologies in C9orf72 amyotrophic lateral sclerosis. Cell Rep..

[150] Burkhardt M.F., Martinez F.J., Wright S., Ramos C., Volfson D., Mason M., Garnes J., Dang V., Lievers J., Shoukat-Mumtaz U. (2013). A cellular model for sporadic ALS using patient-derived induced pluripotent stem cells. Mol. Cell. Neurosci..

[151] Almeida S., Zhang Z., Coppola G., Mao W., Futai K., Karydas A., Geschwind M.D., Tartaglia M.C., Gao F., Gianni D. (2012). Induced pluripotent stem cell models of progranulin-deficient frontotemporal dementia uncover specific reversible neuronal defects. Cell Rep..

[152] Lall D., Workman M.J., Sances S., Ondatje B.N., Bell S., Lawless G., Woodbury A., West D., Meyer A., Matlock A. (2025). An organ-chip model of sporadic ALS using iPSC-derived spinal cord motor neurons and an integrated blood-brain-like barrier. Cell Stem Cell.

[153] Goldberg Y.P., Telenius H., Hayden M.R. (1994). The molecular genetics of Huntington’s disease. Curr. Opin. Neurol..

[154] Pringsheim T., Wiltshire K., Day L., Dykeman J., Steeves T., Jette N. (2012). The incidence and prevalence of Huntington’s disease: A systematic review and meta-analysis. Mov. Disord..

[155] Waldvogel H.J., Kim E.H., Tippett L.J., Vonsattel J.P., Faull R.L. (2015). The Neuropathology of Huntington’s Disease. Curr. Top. BehavNeurosci.

[156] Tanigaki W.K., Rossetti M.A., Rocha N.P., Stimming E.F. (2020). Sleep Dysfunction in Huntington’s Disease: Perspectives from Patients. J. Huntingt. Dis..

[157] Estevez-Fraga C., Flower M.D., Tabrizi S.J. (2020). Therapeutic strategies for Huntington’s disease. Curr. Opin. Neurol..

[158] Park I.H., Lerou P.H., Zhao R., Huo H., Daley G.Q. (2008). Generation of human-induced pluripotent stem cells. Nat. Protoc..

[159] Kedaigle A.J., Fraenkel E., Atwal R.S., Wu M., Gusella J.F., E MacDonald M., A Kaye J., Finkbeiner S., Mattis V.B., The HD iPSC Consortium (2020). HD iPSC Consortium: Bioenergetic deficits in Huntington’s disease iPSC-derived neural cells and rescue with glycolytic metabolites. Hum. Mol. Genet..

[160] Bunting E.L., Donaldson J., Cumming S.A., Olive J., Broom E., Miclăuș M., Hamilton J., Tegtmeyer M., Zhao H.T., Brenton J. (2025). Antisense oligonucleotide-mediated MSH3 suppression reduces somatic CAG repeat expansion in Huntington’s disease iPSC-derived striatal neurons. Sci. Transl. Med..

[161] Mattis V.B., Svendsen C.N. (2017). Modeling Huntington׳s disease with patient-derived neurons. Brain Res..

[162] Geater C., Hernandez S., Thompson L., Mattis V.B. (2018). Cellular Models: HD Patient-Derived Pluripotent Stem Cells. Methods Mol. Biol..

[163] Delli Carri A., Onorati M., Castiglioni V., Faedo A., Camnasio S., Toselli M., Biella G., Cattaneo E. (2013). Human pluripotent stem cell differentiation into authentic striatal projection neurons. Stem Cell Rev. Rep..

[164] Delli Carri A., Onorati M., Lelos M.J., Castiglioni V., Faedo A., Menon R., Camnasio S., Vuono R., Spaiardi P., Talpo F. (2013). Developmentally coordinated extrinsic signals drive human pluripotent stem cell differentiation toward authentic DARPP-32+ medium-sized spiny neurons. Development.

[165] HD iPSC Consortium (2012). Induced pluripotent stem cells from patients with Huntington’s disease show CAG-repeat-expansion-associated phenotypes. Cell Stem Cell.

[166] Camnasio S., Delli Carri A., Lombardo A., Grad I., Mariotti C., Castucci A., Rozell B., Lo Riso P., Castiglioni V., Zuccato C. (2012). The first reported generation of several induced pluripotent stem cell lines from homozygous and heterozygous Huntington’s disease patients demonstrates mutation related enhanced lysosomal activity. Neurobiol. Dis..

[167] Reddy P.H., Shirendeb U.P. (2012). Mutant huntingtin, abnormal mitochondrial dynamics, defective axonal transport of mitochondria, and selective synaptic degeneration in Huntington’s disease. Biochim. Acta.

[168] Juopperi T.A., Kim W.R., Chiang C.H., Yu H., Margolis R.L., Ross C.A., Ming G.L., Song H. (2012). Astrocytes generated from patient induced pluripotent stem cells recapitulate features of Huntington’s disease patient cells. Mol. Brain.

[169] Lisowski P., Lickfett S., Rybak-Wolf A., Menacho C., Le S., Pentimalli T.M., Notopoulou S., Dykstra W., Oehler D., López-Calcerrada S. (2024). Mutant huntingtin impairs neurodevelopment in human brain organoids through CHCHD2-mediated neurometabolic failure. Nat. Commun..

[170] Wu S., Hong Y., Chu C., Gan Y., Li X., Tao M., Wang D., Hu H., Zheng Z., Zhu Q. (2024). Construction of human 3D striato-nigral assembloids to recapitulate medium spiny neuronal projection defects in Huntington’s disease. Proc. Natl. Acad. Sci. USA.

[171] Xu X., Tay Y., Sim B., Yoon S.I., Huang Y., Ooi J., Utami K.H., Ziaei A., Ng B., Radulescu C. (2017). Reversal of Phenotypic Abnormalities by CRISPR/Cas9-Mediated Gene Correction in Huntington Disease Patient-Derived Induced Pluripotent Stem Cells. Stem Cell Rep..

[172] Nekrasov E.D., Vigont V.A., Klyushnikov S.A., Lebedeva O.S., Vassina E.M., Bogomazova A.N., Chestkov I.V., Semashko T.A., Kiseleva E., Suldina L.A. (2016). Manifestation of Huntington’s disease pathology in human induced pluripotent stem cell-derived neurons. Mol. Neurodegener..

[173] Buijsen R.A.M., Hu M., Sáez-González M., Notopoulou S., Mina E., Koning W., Gardiner S.L., van der Graaf L.M., Daoutsali E., Pepers B.A. (2023). Spinocerebellar Ataxia Type 1 Characteristics in Patient-Derived Fibroblast and iPSC-Derived Neuronal Cultures. Mov. Disord..

[174] Fagan K.J., Chillon G., Carrell E.M., Waxman E.A., Davidson B.L. (2024). Cas9 editing of *ATXN1* in a spinocerebellar ataxia type 1 mice and human iPSC-derived neurons. Mol. Ther. Nucleic Acids.

[175] Corti A., Sota R., Dugo M., Calogero R.A., Terragni B., Mantegazza M., Franceschetti S., Restelli M., Gasparini P., Lecis D. (2019). DNA damage and transcriptional regulation in iPSC-derived neurons from Ataxia Telangiectasia patients. Sci. Rep..

[176] Cook S.L., Mensa T., Noma H., Jahnke M., Vidal-Folch N., Stout C., Hrstka R.F., Hrstka S.C.L., Kirkeby L., Oglesbee D. (2026). Clinically discordant siblings with spinal muscular atrophy: Insights from their patient-specific iPSC-derived motor neurons and literature review. NeuromusculDisord.

[177] Lin X., Li J.J., Qian W.J., Zhang Q.J., Wang Z.F., Lu Y.Q., Dong E.L., He J., Wang N., Ma L.X. (2017). Modeling the differential phenotypes of spinal muscular atrophy with high-yield generation of motor neurons from human induced pluripotent stem cells. Oncotarget.

[178] Varderidou-Minasian S., Verheijen B.M., Harschnitz O., Kling S., Karst H., van der Pol W.L., Pasterkamp R.J., Altelaar M. (2021). Spinal Muscular Atrophy Patient iPSC-Derived Motor Neurons Display Altered Proteomes at Early Stages of Differentiation. ACS Omega.

[179] Perego S., Alari V., Pietra G., Lamperti A., Vimercati A., Camporeale N., Garzo M., Cogliati F., Milani D., Vignoli A. (2022). Modeling RTT Syndrome by iPSC-Derived Neurons from Male and Female Patients with Heterogeneously Severe Hot-Spot MECP2 Variants. Int. J. Mol. Sci..

[180] Andoh-Noda T., Inouye M.O., Miyake K., Kubota T., Okano H., Akamatsu W. (2016). Modeling Rett Syndrome Using Human Induced Pluripotent Stem Cells. CNS Neurol. Disord. Drug Targets.

[181] Varderidou-Minasian S., Hinz L., Hagemans D., Posthuma D., Altelaar M., Heine V.M. (2020). Quantitative proteomic analysis of Rett iPSC-derived neuronal progenitors. Mol. Autism.

[182] Doers M.E., Musser M.T., Nichol R., Berndt E.R., Baker M., Gomez T.M., Zhang S.C., Abbeduto L., Bhattacharyya A. (2014). IPSC-derived forebrain neurons from FXS individuals show defects in initial neurite outgrowth. Stem Cells Dev..

[183] Liu X.S., Wu H., Krzisch M., Wu X., Graef J., Muffat J., Hnisz D., Li C.H., Yuan B., Xu C. (2018). Rescue of Fragile X Syndrome Neurons by DNA Methylation Editing of the FMR1 Gene. Cell.

[184] Lu L., Sarkar A.K., Dao L., Liu Y., Ma C., Thwin P.H., Chang X., Yoshida G., Li A., Wang C. (2024). An iPSC model of fragile X syndrome reflects clinical phenotypes and reveals m ^6^ A- mediated epi-transcriptomic dysregulation underlying synaptic dysfunction. bioRxiv.

[185] Russo F.B., Brito A., de Freitas A.M., Castanha A., de Freitas B.C., Beltrão-Braga P.C.B. (2019). The use of iPSC technology for modeling Autism Spectrum Disorders. Neurobiol. Dis..

[186] Hohmann S.S., Ilieva M., Michel T.M. (2020). In vitro models for ASD-patient-derived iPSCs and cerebral organoids. Prog. Mol. Biol. Transl. Sci..

[187] Michels S., Mali A., Jäntti H., Rezaie M., Malm T. (2025). Microglial involvement in autism spectrum disorder: Insights from human data and iPSC models. Brain Behav. Immun..

[188] van der Kant R., Langness V.F., Herrera C.M., Williams D.A., Fong L.K., Leestemaker Y., Steenvoorden E., Rynearson K.D., Brouwers J.F., Helms J.B. (2019). Cholesterol Metabolism Is a Druggable Axis that Independently Regulates Tau and Amyloid-β in iPSC-Derived Alzheimer’s Disease Neurons. Cell Stem Cell.

[189] Kondo T., Imamura K., Funayama M., Tsukita K., Miyake M., Ohta A., Woltjen K., Nakagawa M., Asada T., Arai T. (2017). IPSC-Based Compound Screening and In Vitro Trials Identify a Synergistic Anti-amyloid β Combination for Alzheimer’s Disease. Cell Rep..

[190] Kondo T., Banno H., Okunomiya T., Amino Y., Endo K., Nakakura A., Uozumi R., Kinoshita A., Tada H., Morita S. (2021). Repurposing bromocriptine for Aβ metabolism in Alzheimer’s disease (REBRAnD) study: Randomised placebo-controlled double-blind comparative trial and open-label extension trial to investigate the safety and efficacy of bromocriptine in Alzheimer’s disease with presenilin 1 (PSEN1) mutations. BMJ Open.

[191] Mertens J., Stüber K., Wunderlich P., Ladewig J., Kesavan J.C., Vandenberghe R., Vandenbulcke M., van Damme P., Walter J., Brüstle O. (2013). APP processing in human pluripotent stem cell-derived neurons is resistant to NSAID-based γ-secretase modulation. Stem Cell Rep..

[192] Hung C.O., Livesey F.J. (2018). Altered γ-secretase processing of APP disrupts lysosome and autophagosome function in monogenic Alzheimer’s disease. Cell Rep..

[193] Liu Q., Waltz S., Woodruff G., Ouyang J., Israel M.A., Herrera C., Sarsoza F., Tanzi R.E., Koo E.H., Ringman J.M. (2014). Effect of potent γ-secretase modulator in human neurons derived from multiple presenilin 1–induced pluripotent stem cell mutant carriers. JAMA Neurol..

[194] Chang C.-Y., Chen S.-M., Lu H.-E., Lai S.-M., Lai P.-S., Shen P.-W., Chen P.-Y., Shen C.-I., Harn H.-J., Lin S.-Z. (2015). N-butylidenephthalide attenuates Alzheimer’s disease-like cytopathy in Down syndrome induced pluripotent stem cell-derived neurons. Sci. Rep..

[195] Balez R., Steiner N., Engel M., Muñoz S.S., Lum J.S., Wu Y., Wang D., Vallotton P., Sachdev P., O’connor M. (2016). Neuroprotective effects of apigenin against inflammation, neuronal excitability and apoptosis in an induced pluripotent stem cell model of Alzheimer’s disease. Sci. Rep..

[196] Yamaguchi A., Ishikawa K.I., Inoshita T., Shiba-Fukushima K., Saiki S., Hatano T., Mori A., Oji Y., Okuzumi A., Li Y. (2020). Identifying Therapeutic Agents for Amelioration of Mitochondrial Clearance Disorder in Neurons of Familial Parkinson Disease. Stem Cell Rep..

[197] Jarazo J., Barmpa K., Modamio J., Saraiva C., Sabaté-Soler S., Rosety I., Griesbeck A., Skwirblies F., Zaffaroni G., Smits L.M. (2022). Parkinson’s Disease Phenotypes in Patient Neuronal Cultures and Brain Organoids Improved by 2-Hydroxypropyl-β-Cyclodextrin Treatment. Mov. Disord..

[198] Kim M.S., Ra E.A., Kweon S.H., Seo B.A., Ko H.S., Oh Y., Lee G. (2023). Advanced human iPSC-based preclinical model for Parkinson’s disease with optogenetic alpha-synuclein aggregation. Cell Stem Cell.

[199] Sahin G.S., Guyett P.J., Xu K., Kouznetsova J., Zheng W., Hendrickson M. (2026). High-throughput screening of ALS patient iPSC-derived spinal motor neurons identifies novel compounds that increase neurofilament light chain expression. SLAS Discov..

[200] Czuppa M., Dhingra A., Zhou Q., Schludi C., König L., Scharf E., Farny D., Dalmia A., Täger J., Castillo-Lizardo M. (2022). Drug screen in iPSC-Neurons identifies nucleoside analogs as inhibitors of (G_4_C_2_)_n_ expression in C9orf72 ALS/FTD. Cell Rep..

[201] Castillo Bautista C.M., Eismann K., Gentzel M., Pelucchi S., Mertens J., Walters H.E., Yun M.H., Sterneckert J. (2023). Obatoclax Rescues FUS-ALS Phenotypes in iPSC-Derived Neurons by Inducing Autophagy. Cells.

[202] Bye C.R., Qian E., Lim K., Daniszewski M., Garton F.C., Trần-Lê B.C., Liang H.H., Lin T., Lock J.G., Crombie D.E. (2026). Large-scale drug screening in iPSC-derived motor neurons from sporadic ALS patients identifies a potential combinatorial therapy. Nat. Neurosci..

[203] Armijo E., Edwards G., Flores A., Vera J., Shahnawaz M., Moda F., Gonzalez C., Sanhueza M., Soto C. (2021). Induced Pluripotent Stem Cell-Derived Neural Precursors Improve Memory, Synaptic and Pathological Abnormalities in a Mouse Model of Alzheimer’s Disease. Cells.

[204] Kikuchi T., Morizane A., Doi D., Magotani H., Onoe H., Hayashi T., Mizuma H., Takara S., Takahashi R., Inoue H. (2017). Human iPS cell-derived dopaminergic neurons function in a primate Parkinson’s disease model. Nature.

[205] Fu C.L., Dong B.C., Jiang X., Li D., Yao J. (2024). A cell therapy approach based on iPSC-derived midbrain organoids for the restoration of motor function in a Parkinson’s disease mouse model. Heliyon.

[206] Hiller B.M., Marmion D.J., Thompson C.A., Elliott N.A., Federoff H., Brundin P., Mattis V.B., McMahon C.W., Kordower J.H. (2022). Optimizing maturity and dose of iPSC-derived dopamine progenitor cell therapy for Parkinson’s disease. npj Regen. Med..

[207] Kondo T., Funayama M., Tsukita K., Hotta A., Yasuda A., Nori S., Kaneko S., Nakamura M., Takahashi R., Okano H. (2014). Focal transplantation of human iPSC-derived glial-rich neural progenitors improves lifespan of ALS mice. Stem Cell Rep..

[208] Knippenberg S., Rath K.J., Böselt S., Thau-Habermann N., Schwarz S.C., Dengler R., Wegner F., Petri S. (2017). Intraspinal administration of human spinal cord-derived neural progenitor cells in the G93A-SOD1 mouse model of ALS delays symptom progression, prolongs survival and increases expression of endogenous neurotrophic factors. J. Tissue Eng. Regen. Med..

[209] Popescu I.R., Nicaise C., Liu S., Bisch G., Knippenberg S., Daubie V., Bohl D., Pochet R. (2013). Neural progenitors derived from human induced pluripotent stem cells survive and differentiate upon transplantation into a rat model of amyotrophic lateral sclerosis. Stem Cells Transl. Med..

[210] Osipovitch M., Asenjo Martinez A., Mariani J.N., Cornwell A., Dhaliwal S., Zou L., Chandler-Militello D., Wang S., Li X., Benraiss S.J. (2019). Human ESC-Derived Chimeric Mouse Models of Huntington’s Disease Reveal Cell-Intrinsic Defects in Glial Progenitor Cell Differentiation. Cell Stem Cell.

[211] Choi K.A., Choi Y., Hong S. (2018). Stem cell transplantation for Huntington’s diseases. Methods.

[212] Cho I.K., Yang B., Forest C., Qian L., Chan A.W.S. (2019). Amelioration of Huntington’s disease phenotype in astrocytes derived from iPSC-derived neural progenitor cells of Huntington’s disease monkeys. PLoS ONE.

[213] Takahashi J. (2020). IPS cell-based therapy for Parkinson’s disease: A Kyoto trial. Regen. Ther..

[214] Piao J., Zabierowski S., Dubose B.N., Hill E.J., Navare M., Claros N., Rosen S., Ramnarine K., Horn C., Fredrickson C. (2021). Preclinical Efficacy and Safety of a Human Embryonic Stem Cell-Derived Midbrain Dopamine Progenitor Product, MSK-DA01. Cell Stem Cell.

[215] Schweitzer J.S., Song B., Herrington T.M., Park T.Y., Lee N., Ko S., Jeon J., Cha Y., Kim K., Li Q. (2020). Personalized iPSC-Derived Dopamine Progenitor Cells for Parkinson’s Disease. N. Engl. J. Med..

[216] Gotkine M., Caraco Y., Lerner Y., Blotnick S., Wanounou M., Slutsky S.G., Chebath J., Kuperstein G., Estrin E., Ben-Hur T. (2023). Safety and efficacy of first-in-man intrathecal injection of human astrocytes (AstroRx®) in ALS patients: Phase I/IIa clinical trial results. J. Transl. Med..

[217] Shayestehfar M., Taherkhani T., Jahandideh P., Hamidieh A.A., Faramarzpour M., Memari A. (2025). Brain tumors and induced pluripotent stem cell technology: A systematic review of the literature. Ann. Med. Surg..

[218] Mertens J., Reid D., Lau S., Kim Y., Gage F.H. (2018). Aging in a Dish: IPSC-Derived and Directly Induced Neurons for Studying Brain Aging and Age-Related Neurodegenerative Diseases. Annu. Rev. Genet..

[219] Traxler L., Edenhofer F., Mertens J. (2019). Next-generation disease modeling with direct conversion: A new path to old neurons. FEBS Lett..

[220] Imaizumi K., Sone T., Ibata K., Fujimori K., Yuzaki M., Akamatsu W., Okano H. (2015). Controlling the Regional Identity of hPSC-Derived Neurons to Uncover Neuronal Subtype Specificity of Neurological Disease Phenotypes. Stem Cell Rep..

[221] Victor M.B., Richner M., Olsen H.E., Lee S.W., Monteys A.M., Ma C., Huh C.J., Zhang B., Davidson B.L., Yang X.W. (2018). Striatal neurons directly converted from Huntington’s disease patient fibroblasts recapitulate age-associated disease phenotypes. Nat. Neurosci..

[222] Nwokoye P.N., Abilez O.J. (2024). Bioengineering methods for vascularizing organoids. Cell Rep. Methods.

